# Publisher Correction: Doubly N-confused and ring-contracted [24] hexaphyrin Pd-complexes as stable antiaromatic N-confused expanded porphyrins

**DOI:** 10.1038/s41467-023-41072-5

**Published:** 2023-08-31

**Authors:** Fuying Luo, Le Liu, Han Wu, Ling Xu, Yutao Rao, Mingbo Zhou, Atsuhiro Osuka, Jianxin Song

**Affiliations:** Key Laboratory of Chemical Biology and Traditional Chinese Medicine Research (Ministry of Education of China), Key Laboratory of the Assembly and Application of Organic Functional Molecules of Hunan Province, College of Chemistry and Chemical Engineering, Hunan Normal University, 410081 Changsha, China

**Keywords:** Synthetic chemistry methodology, Chemical bonding, Chemical bonding

Correction to: *Nature Communications* 10.1038/s41467-023-40700-4, published online 18 August 2023

In the original version of this Article the order of Figures 3 to 9 was incorrectly displayed. This has been corrected in both the PDF and HTML versions of the Article.

