# Supplementary material for: Doubly N-confused and ring-contracted [24]hexaphyrin Pd-complexes as stable antiaromatic N-confused expanded porphyrins
Source: Nat Commun. 2023 Aug 18;14:5028. doi: 10.1038/s41467-023-40700-4 (PMC10439157; doi:10.1038/s41467-023-40700-4)
Supplement: Supplementary file 1 — Supplementary Information [file 41467_2023_40700_MOESM1_ESM.pdf]

Supplementary Information for

Doubly N-Confused and Ring-Contracted [24]Hexaphyrin Pd-Complexes: Stable Antiaromatic N-Confused Expanded Porphyrins

Luo and Liu et al.

Supplementary Figures

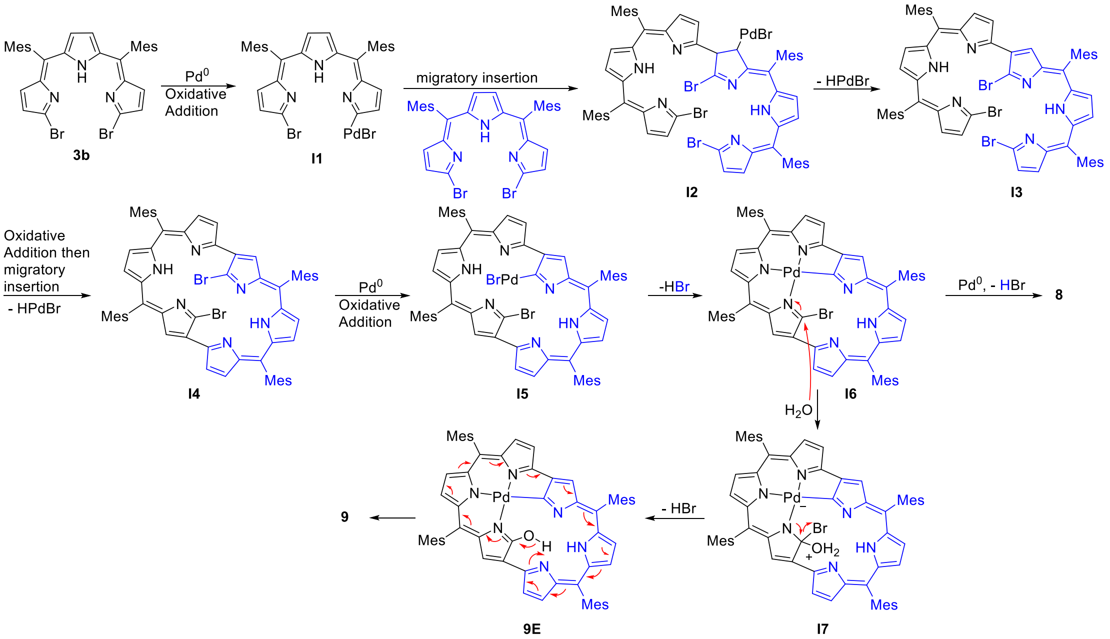

Supplementary Figure 1. Proposed mechanism for the formation of 8 and 9. Oxidation and migratory insertion result in the formation of the skeleton of N-confused hexaphyrins. Following reaction with water leads to the lactam unit.

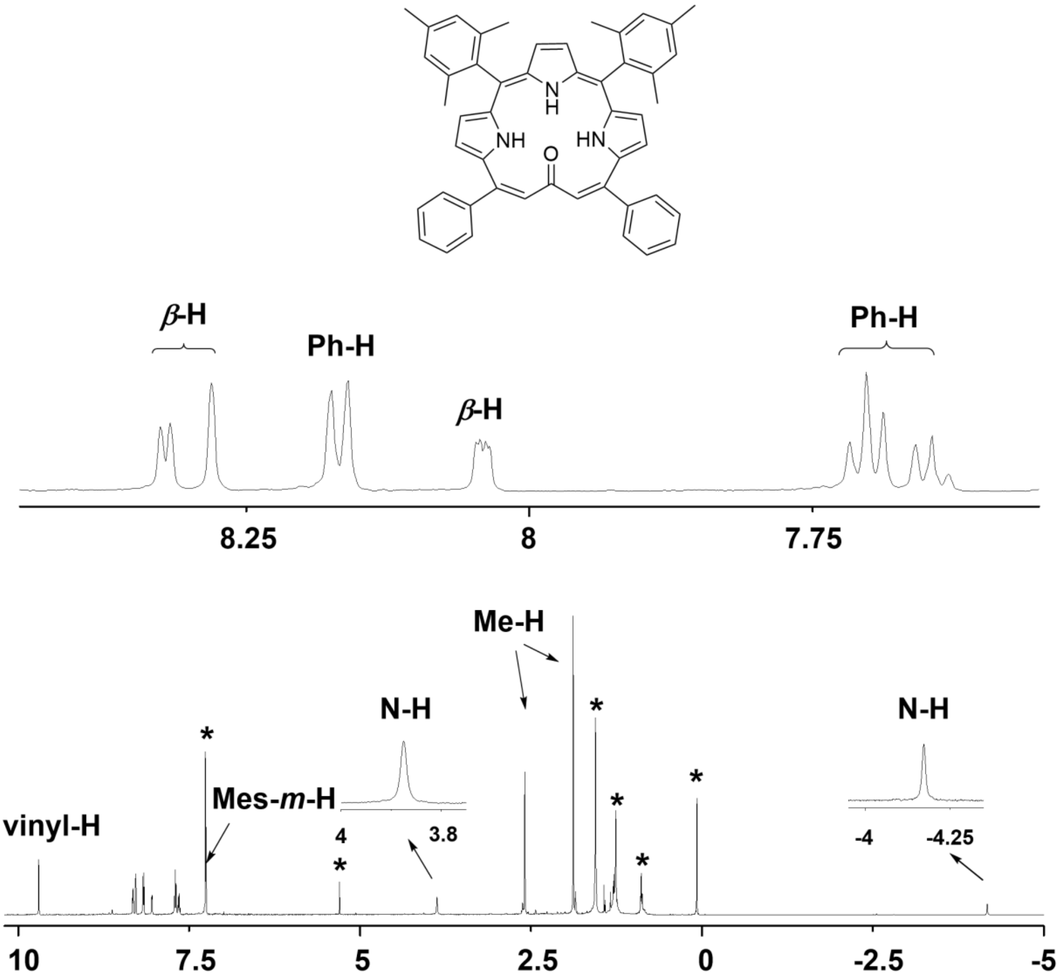

Supplementary Figure 2. ¹H NMR spectrum of 6 in $CDCl_3$. *Solvent or impurities.

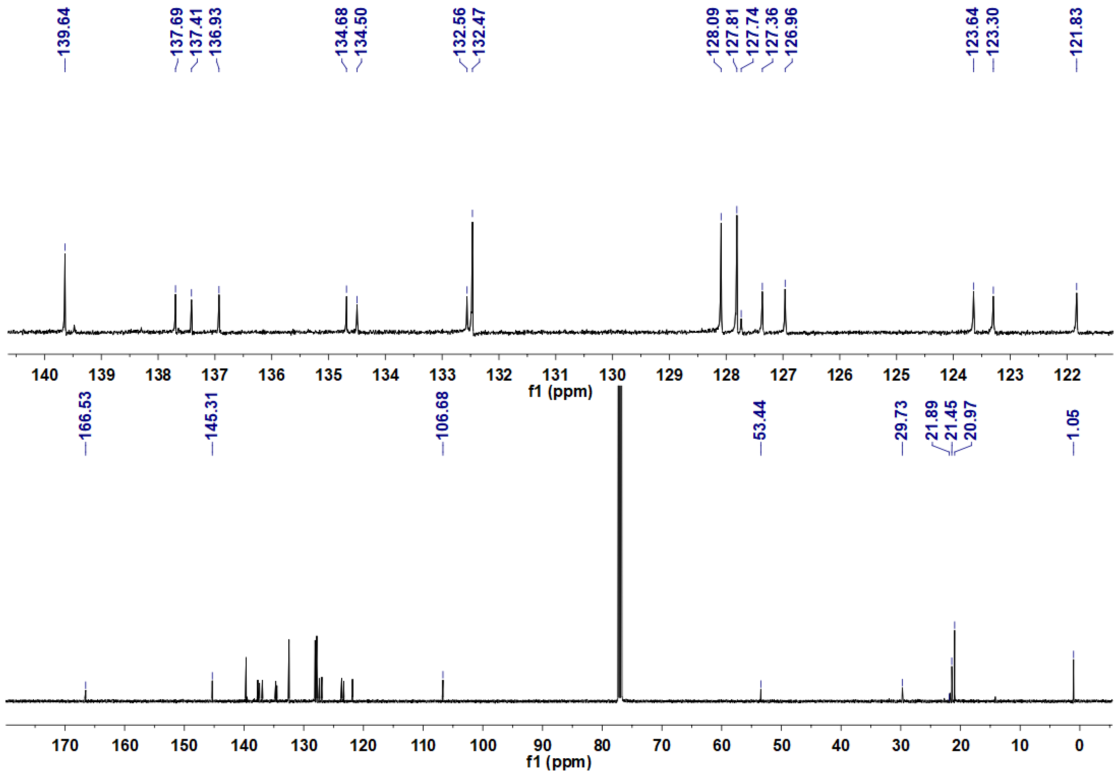

Supplementary Figure 3. ^{13}C NMR spectrum of 6 in CDCl$_3$. The absence of some peaks in ^{13}C NMR may be due to overlapping.

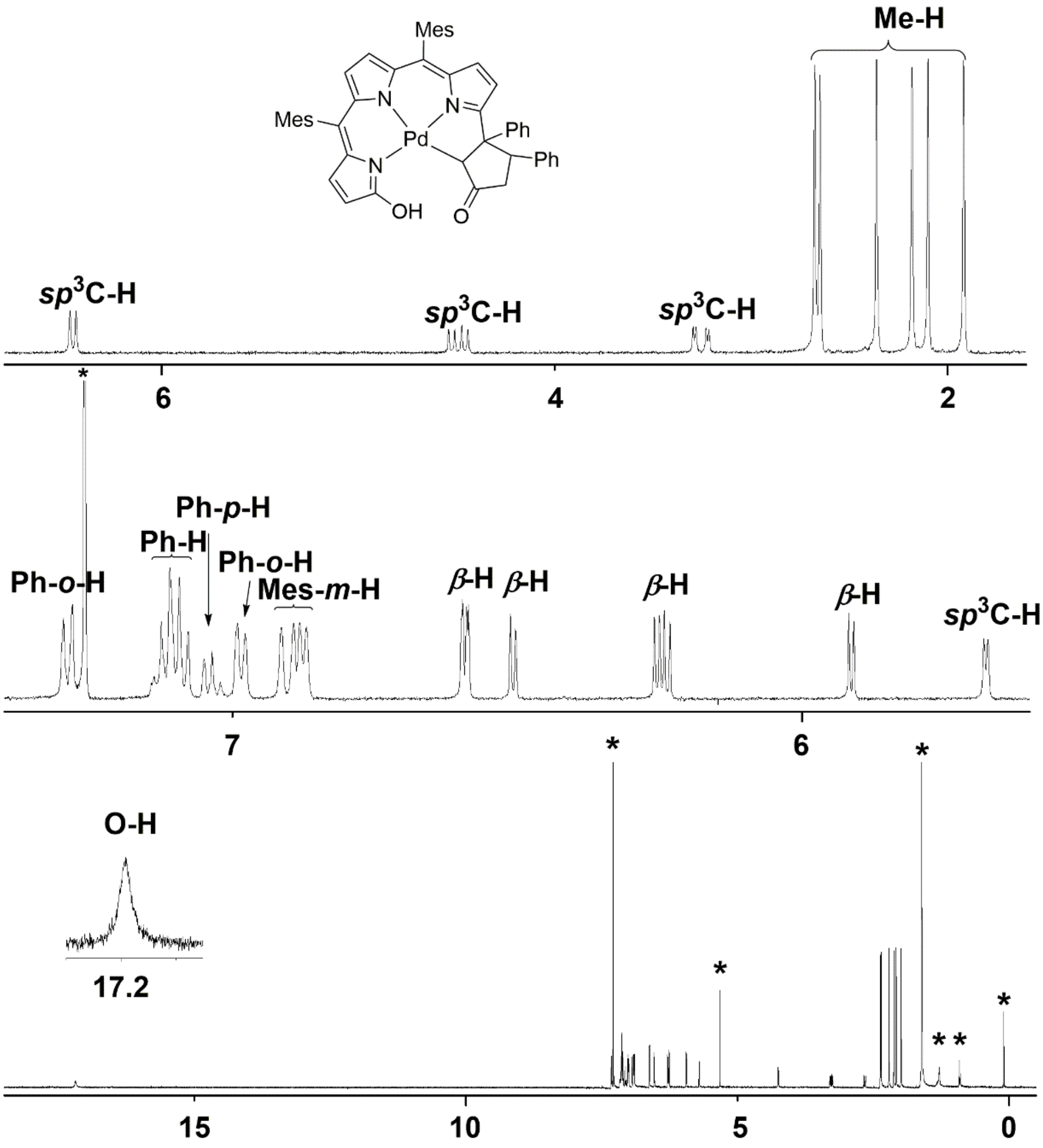

Supplementary Figure 4. ^{1}H NMR spectrum of 7 in CDCl$_3$. *Solvent or impurities.

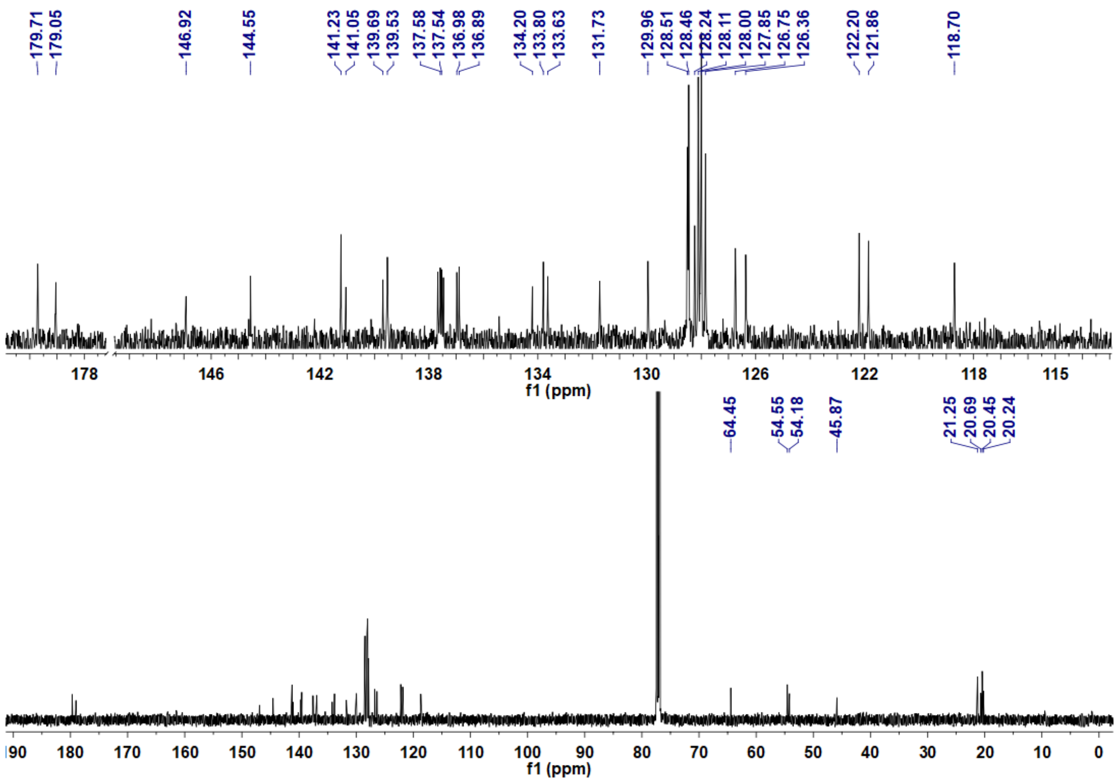

Supplementary Figure 5. ^{13}C NMR spectrum of 7 in $CDCl_3$. The absence of some peaks in ^{13}C NMR may be due to overlapping.

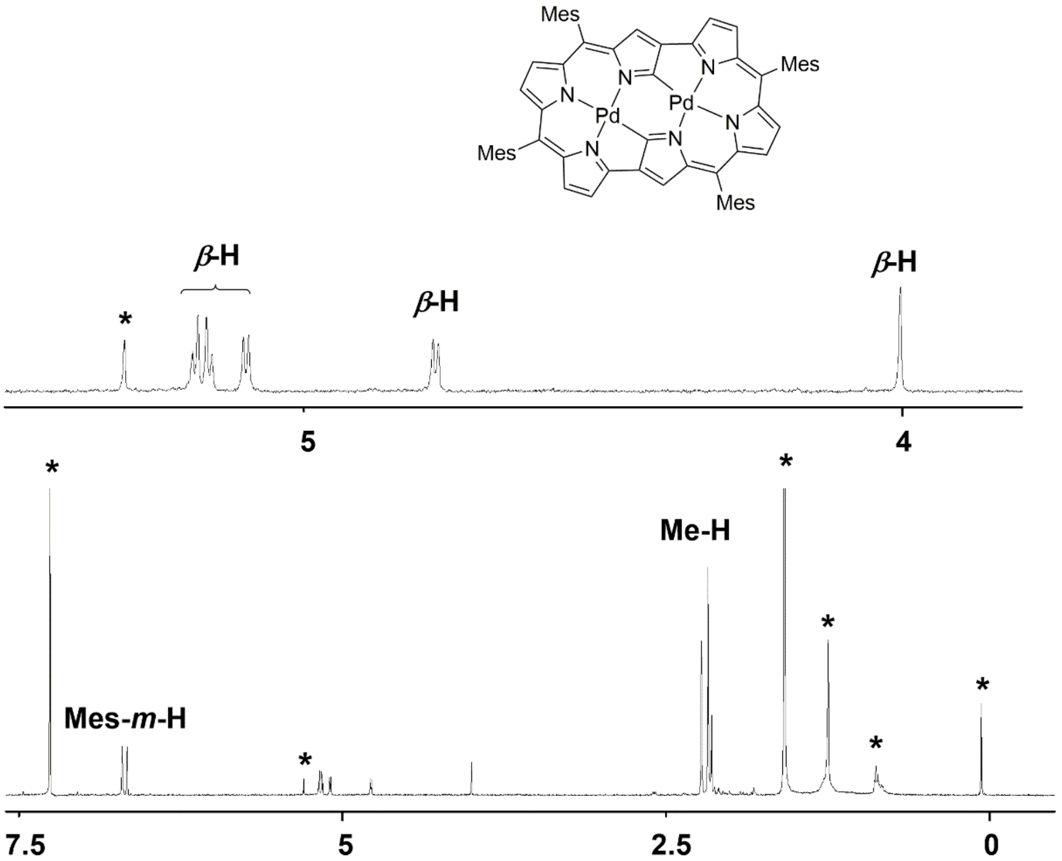

Supplementary Figure 6. ^{1}H NMR spectrum of 8 in $CDCl_3$. *Solvent or impurities.

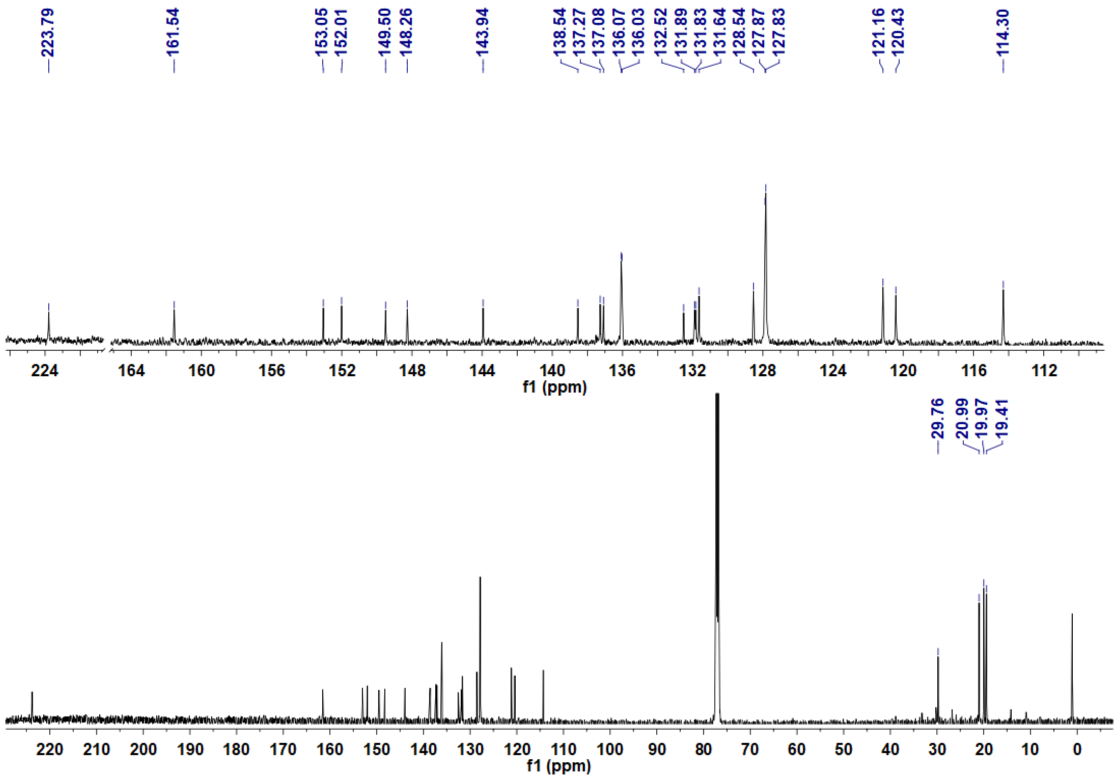

Supplementary Figure 7. ^{13}C NMR spectrum of 8 in $CDCl_3$. The absence of some peaks in ^{13}C NMR may be due to overlapping.

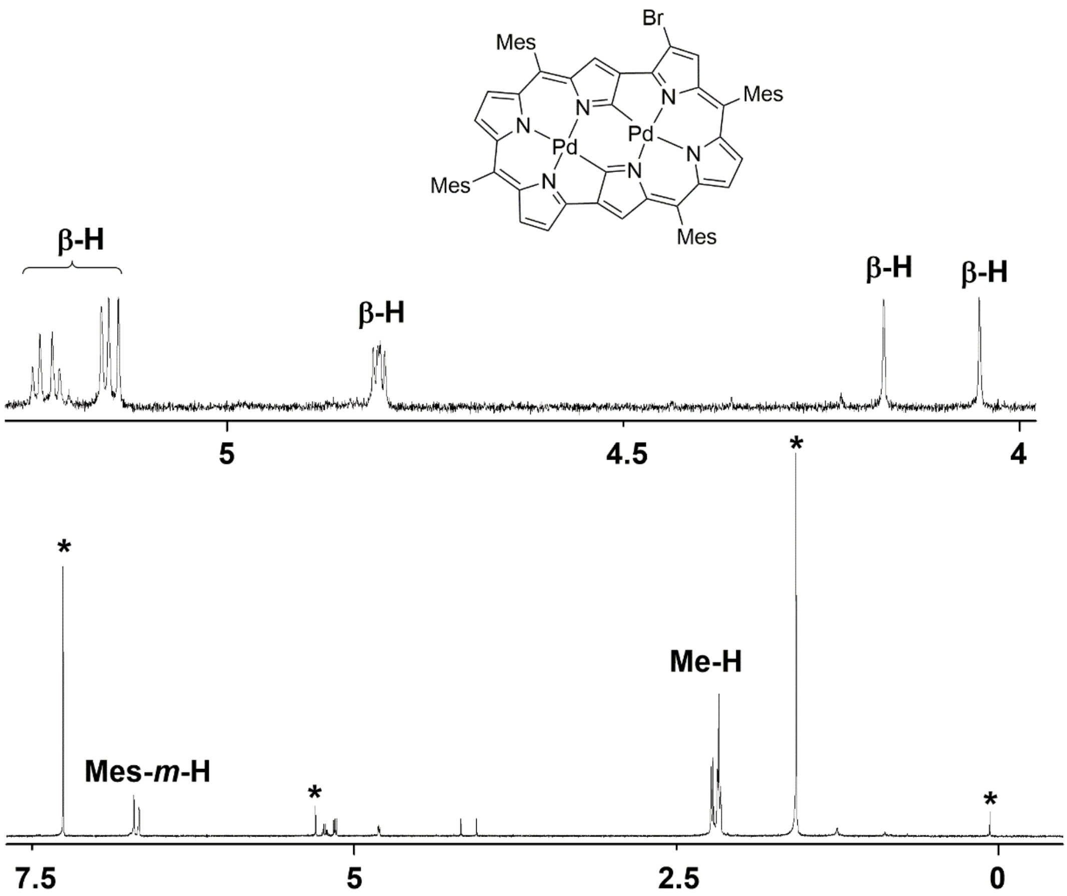

Supplementary Figure 8. ^{1}C NMR spectrum of 8a in $CDCl_3$. *Solvent or impurities.

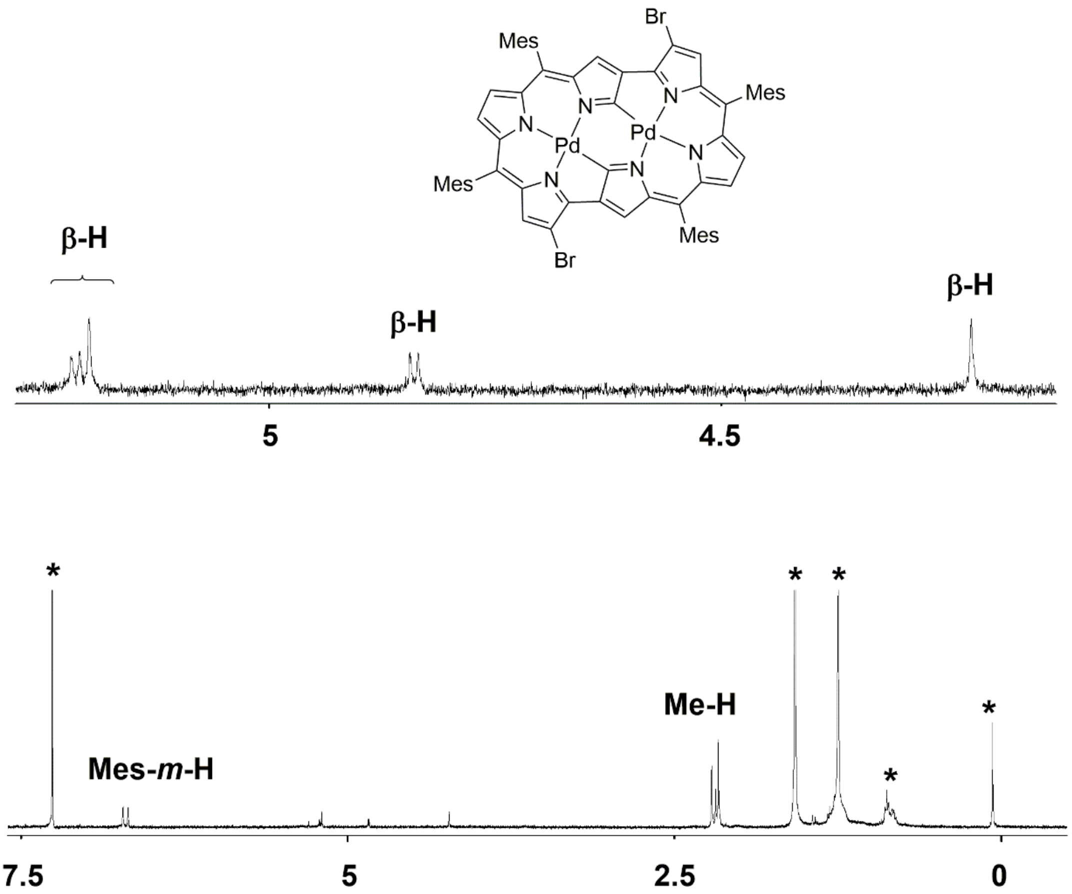

Supplementary Figure 9. ^{1}H NMR spectrum of 8b in $CDCl_3$. *Solvent or impurities.

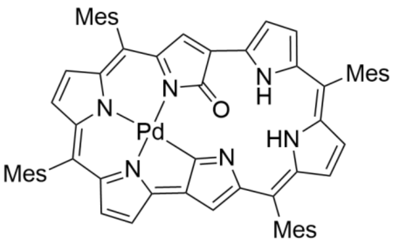

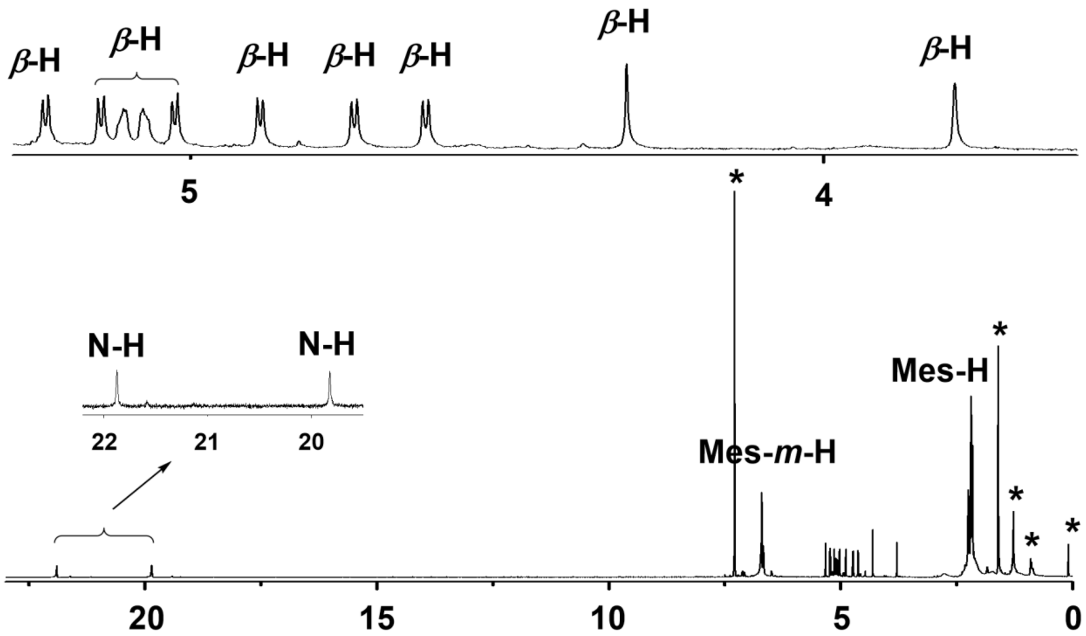

Supplementary Figure 10. ^{1}H NMR spectrum of 9 in CDCl₃. *Solvent or impurities.

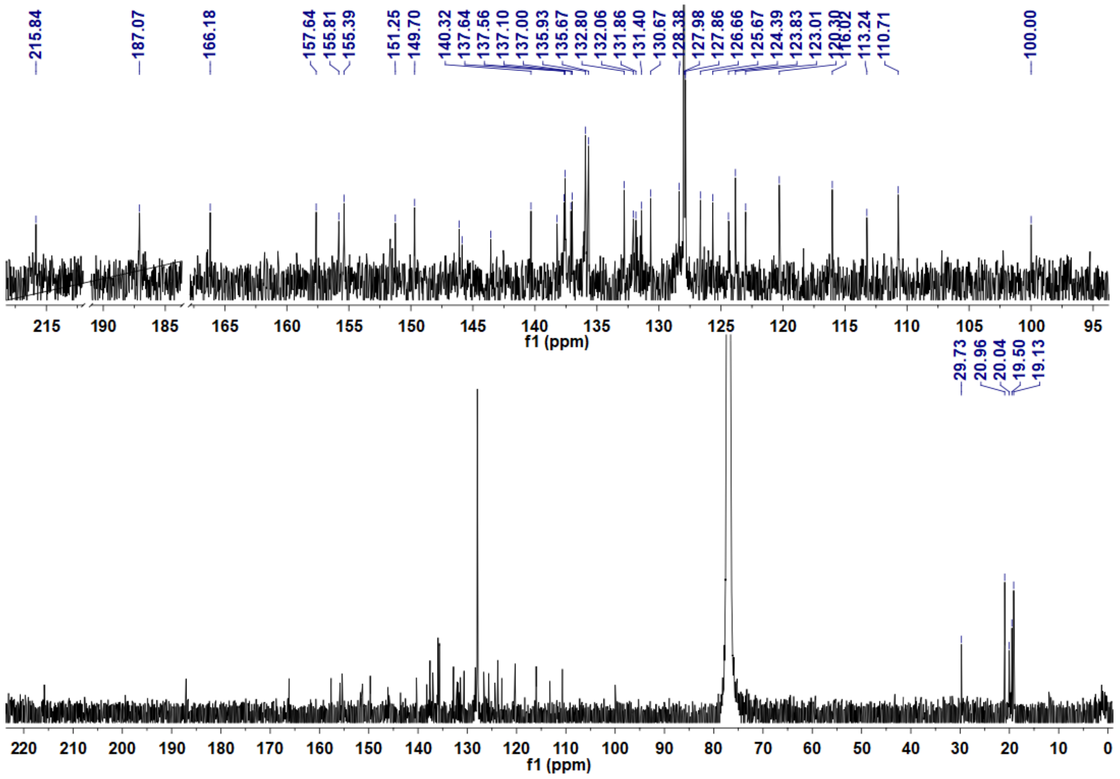

Supplementary Figure 11. ^{13}C NMR spectrum of 9 in $CDCl_3$. The absence of some peaks in ^{13}C NMR may be due to overlapping.

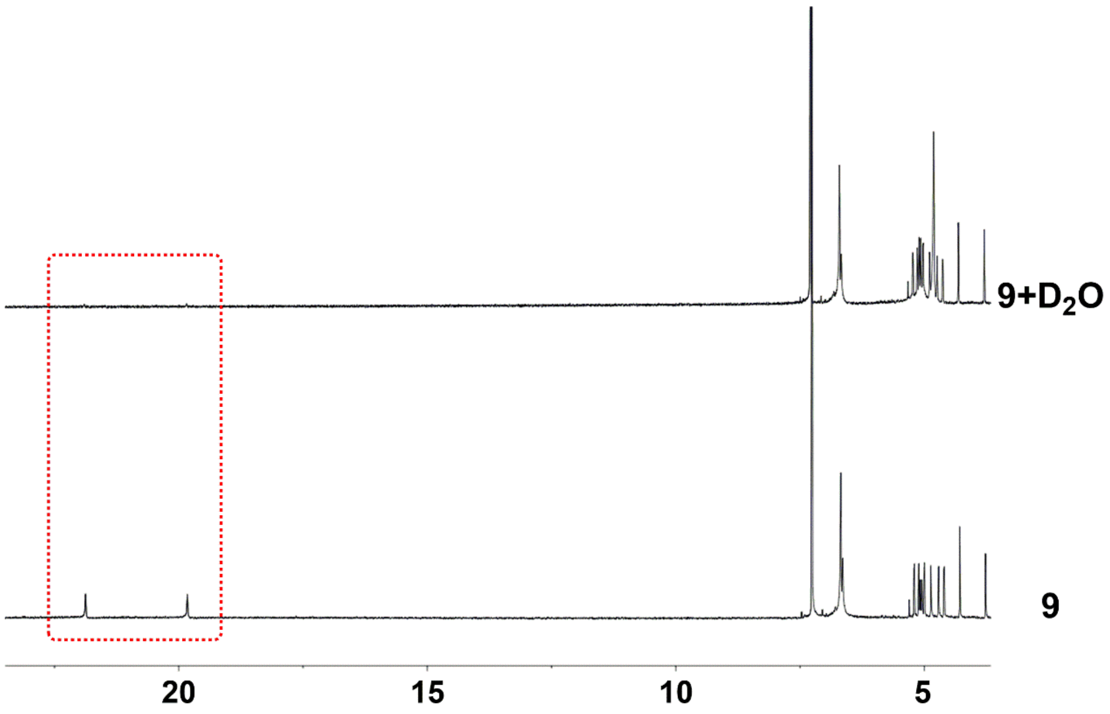

Supplementary Figure 12. ^{1}H NMR spectra of 9 and 9+D$_2$O in CDCl$_3$. The red dotted box indicates the disappearance of NH.

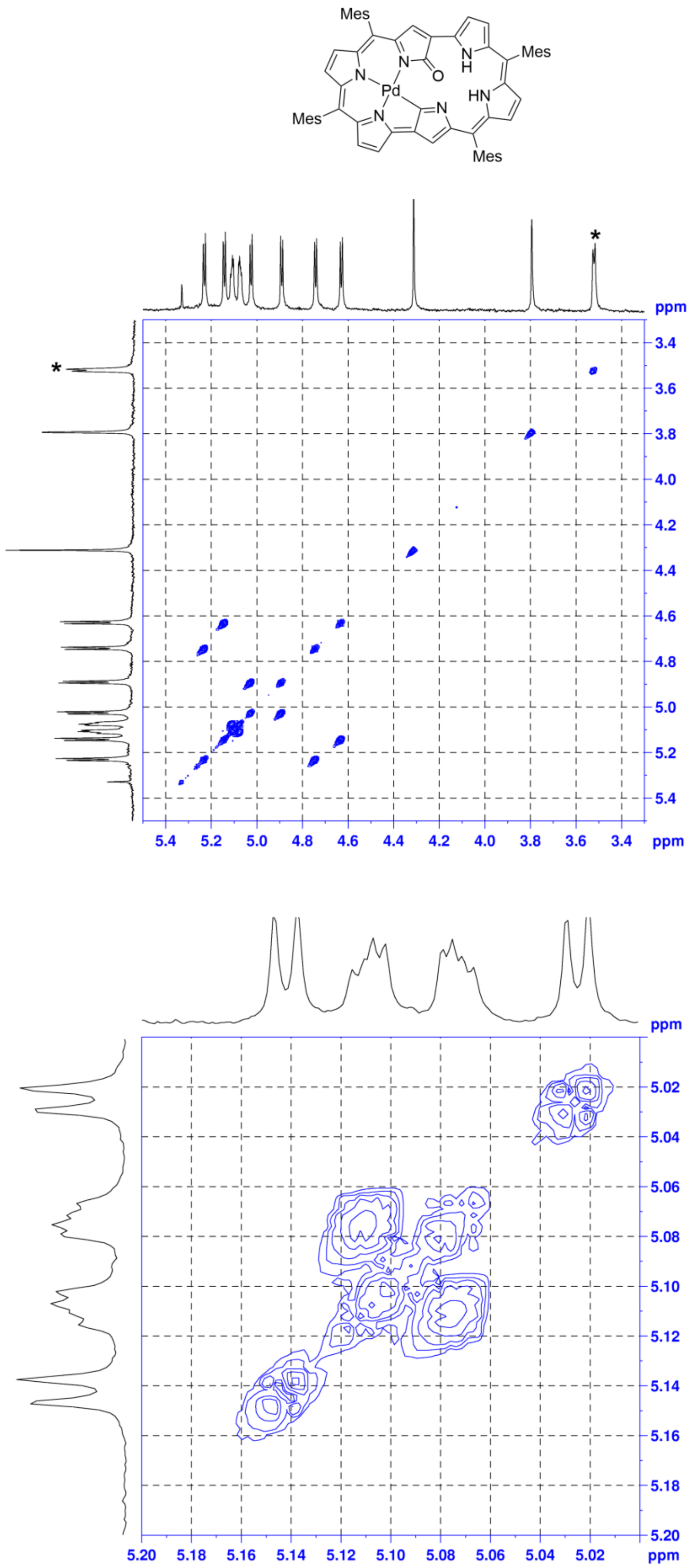

Supplementary Figure 13. **COSY spectrum of 9 in $CDCl_3$.** *Impurities.

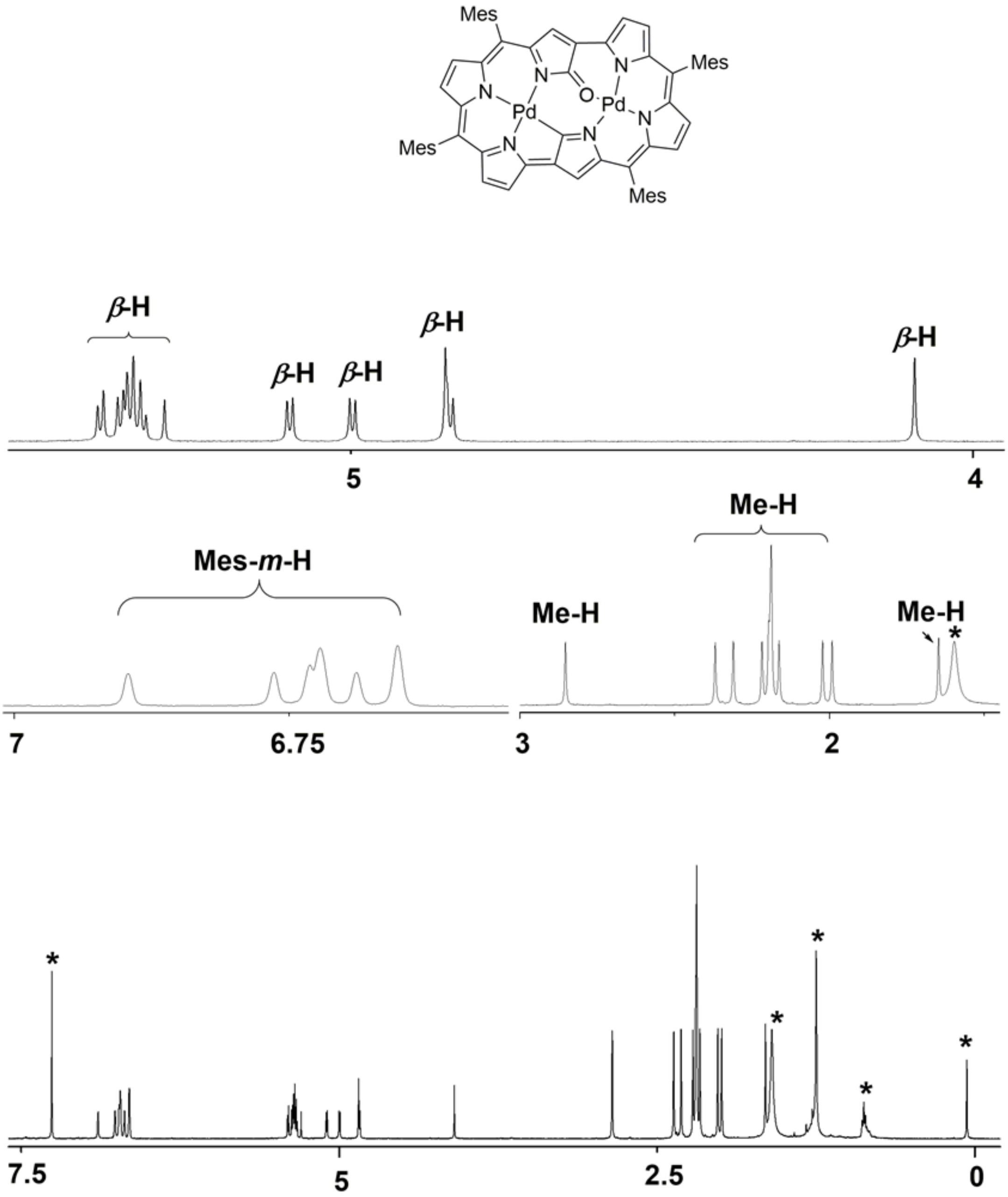

Supplementary Figure 14. ^{1}H NMR spectrum of 9Pd in $CDCl_3$. *Solvent or impurities.

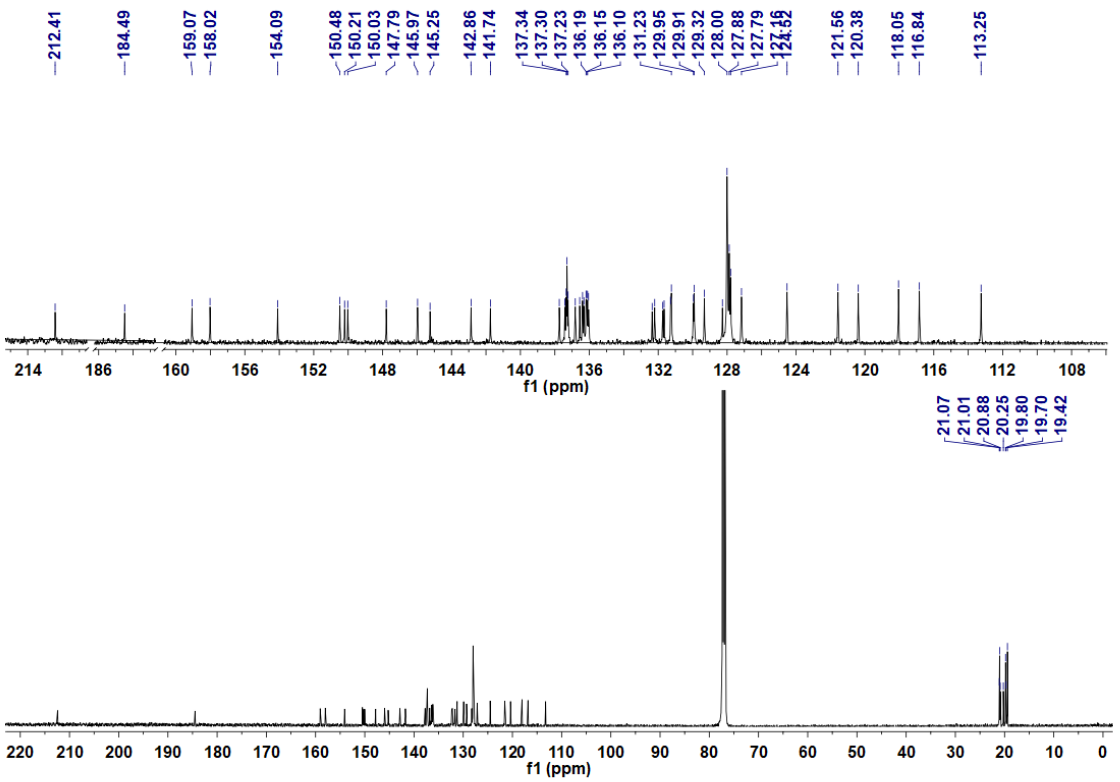

Supplementary Figure 15. ^{13}C NMR spectrum of 9Pd in $CDCl_3$. The absence of some peaks in ^{13}C NMR may be due to overlapping.

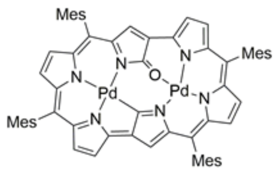

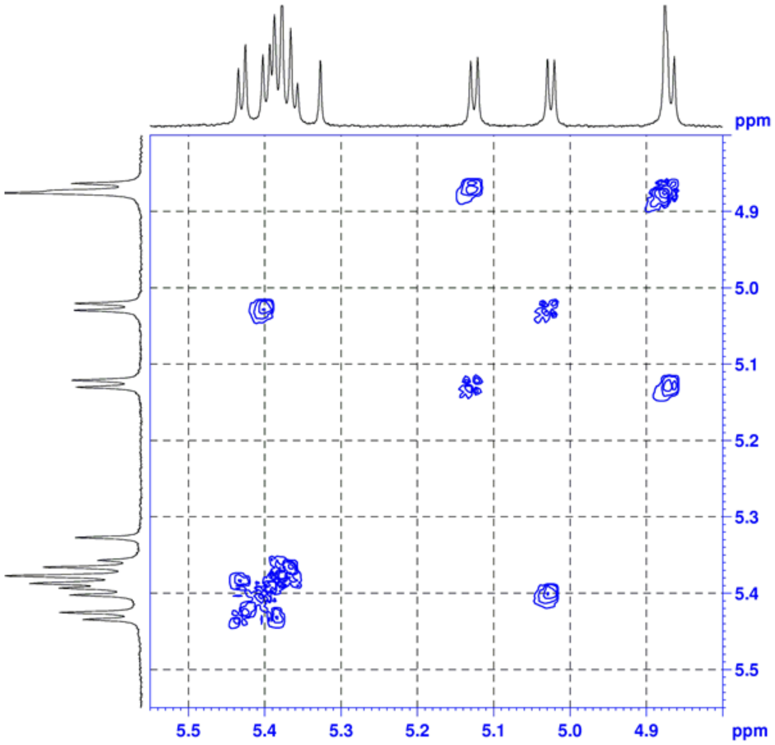

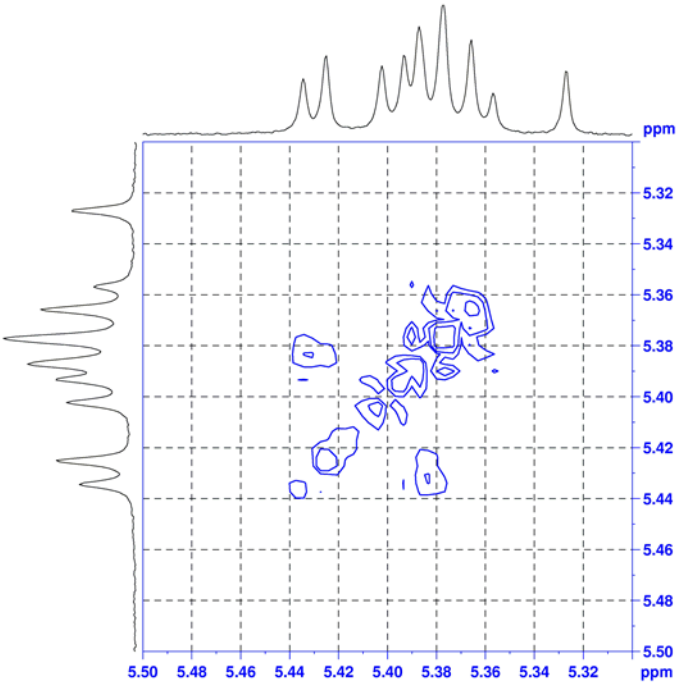

Supplementary Figure 16. **COSY spectrum of 9Pd in $CDCl_3$.** The bottom one is the partial spectrum in the region between 5-30-5.50 ppm.

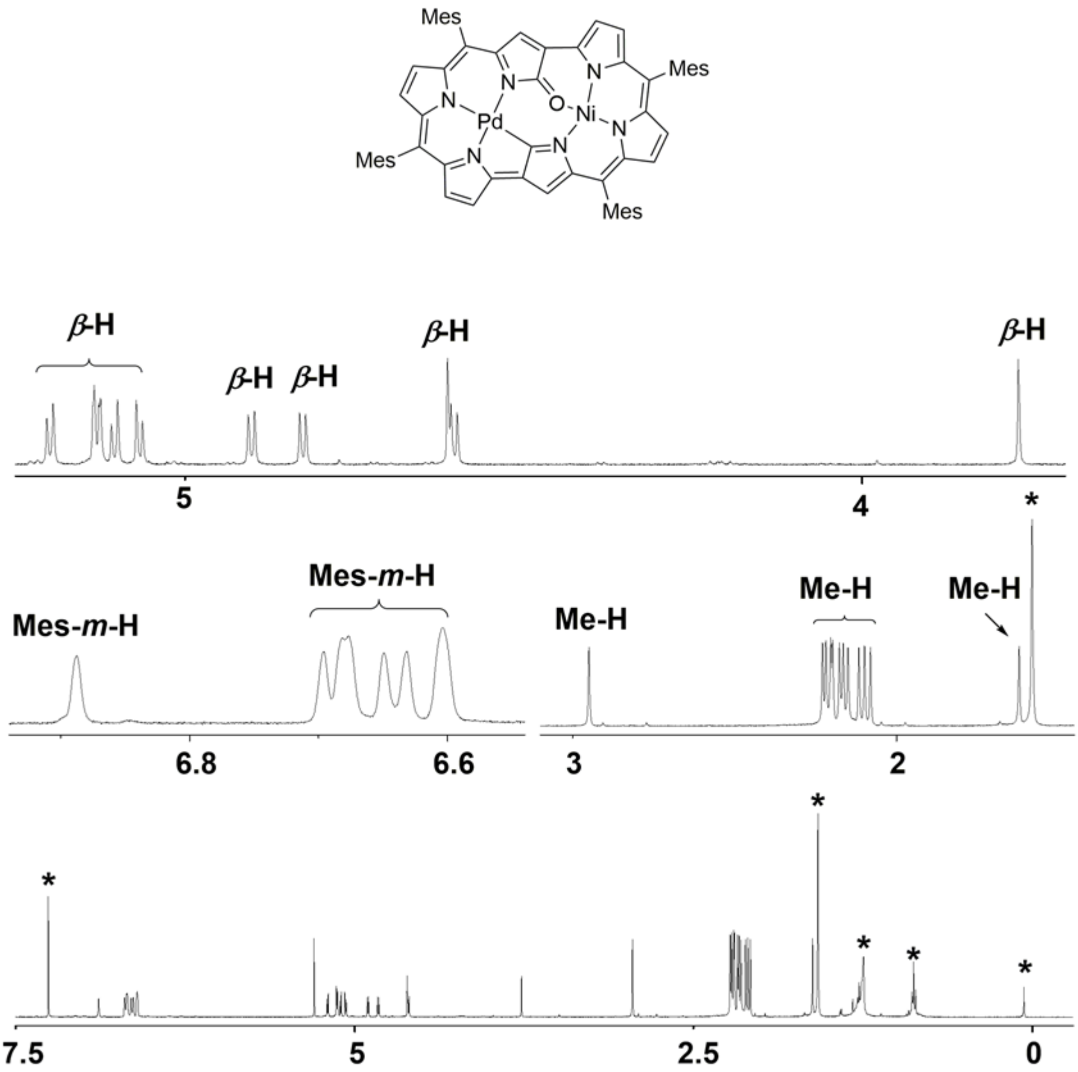

Supplementary Figure 17. ^{1}H NMR spectrum of 9Ni in $CDCl_3$. *Solvent or impurities.

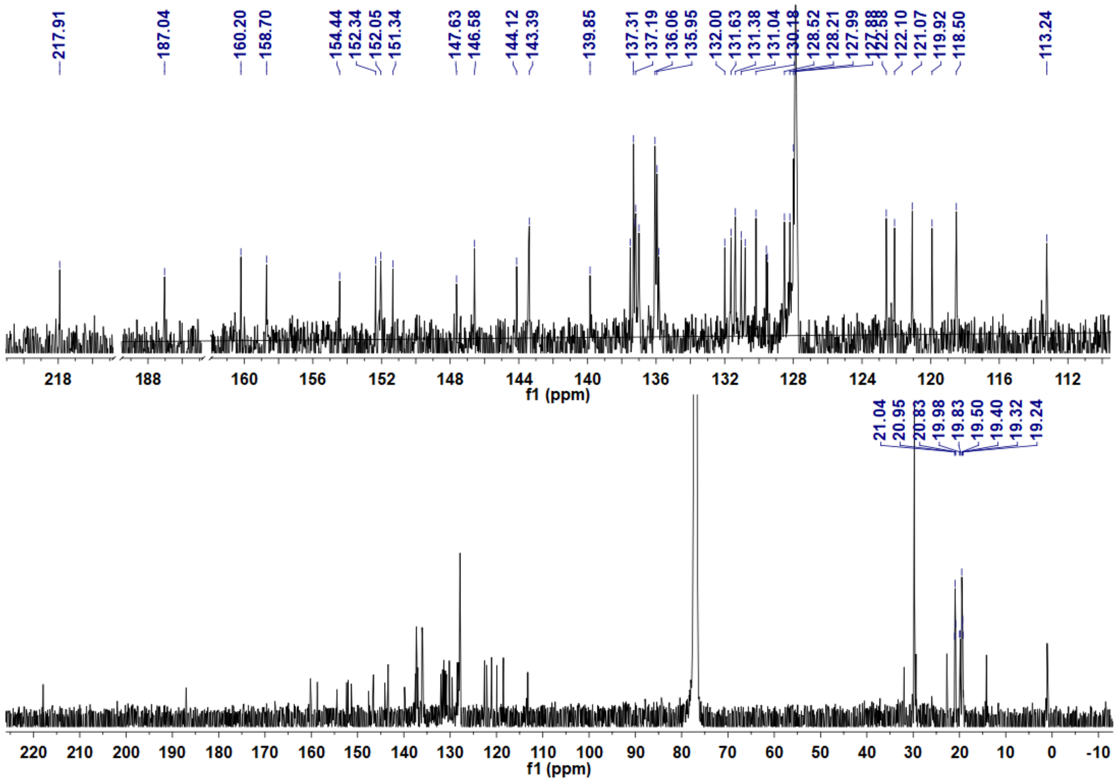

Supplementary Figure 18. ^{13}C NMR spectrum of 9Ni in $CDCl_3$. The absence of some peaks in ^{13}C NMR may be due to overlapping.

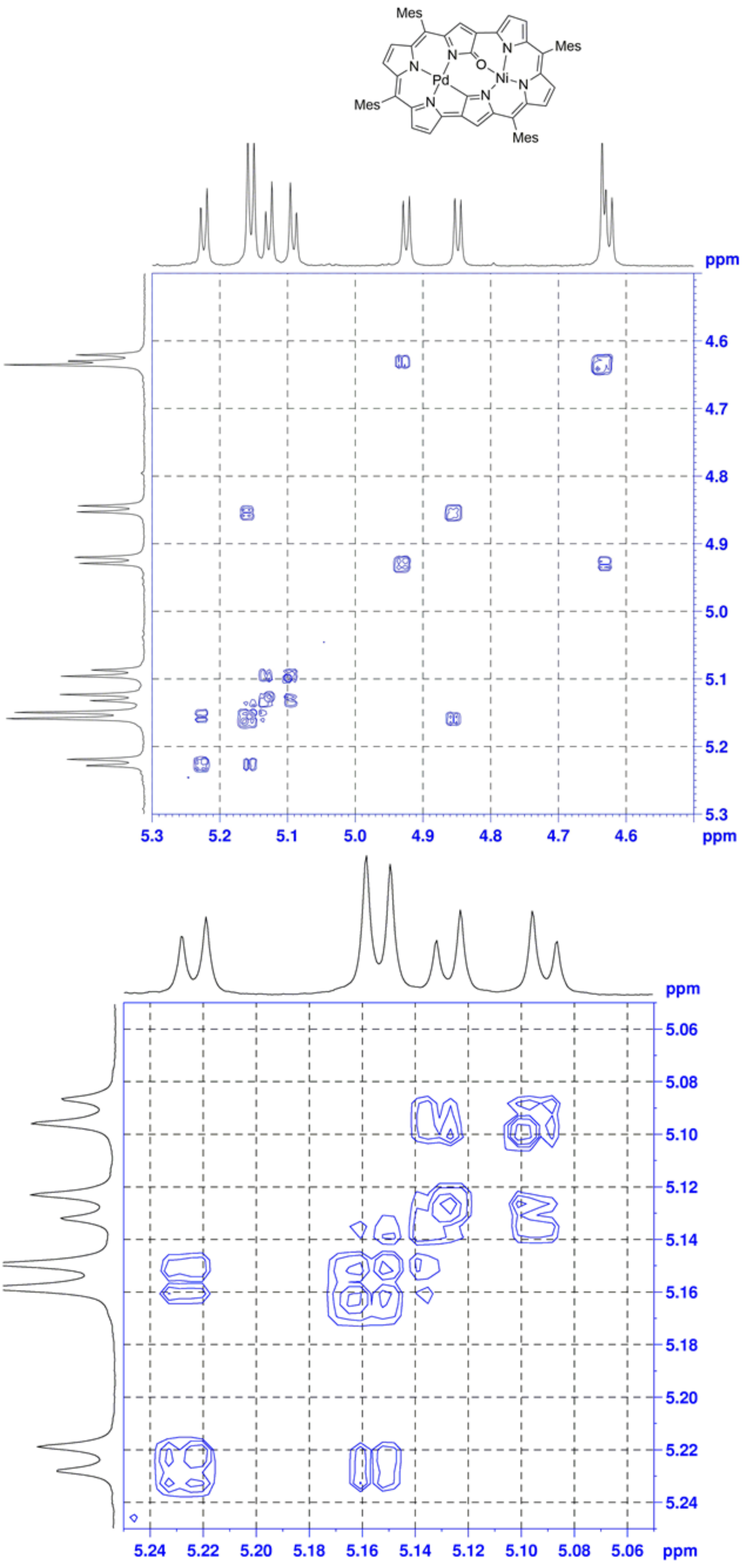

Supplementary Figure 19. **COSY spectrum of 9Ni in $CDCl_3$.** The bottom one is the partial spectrum in the region between 5.05-5.25 ppm.

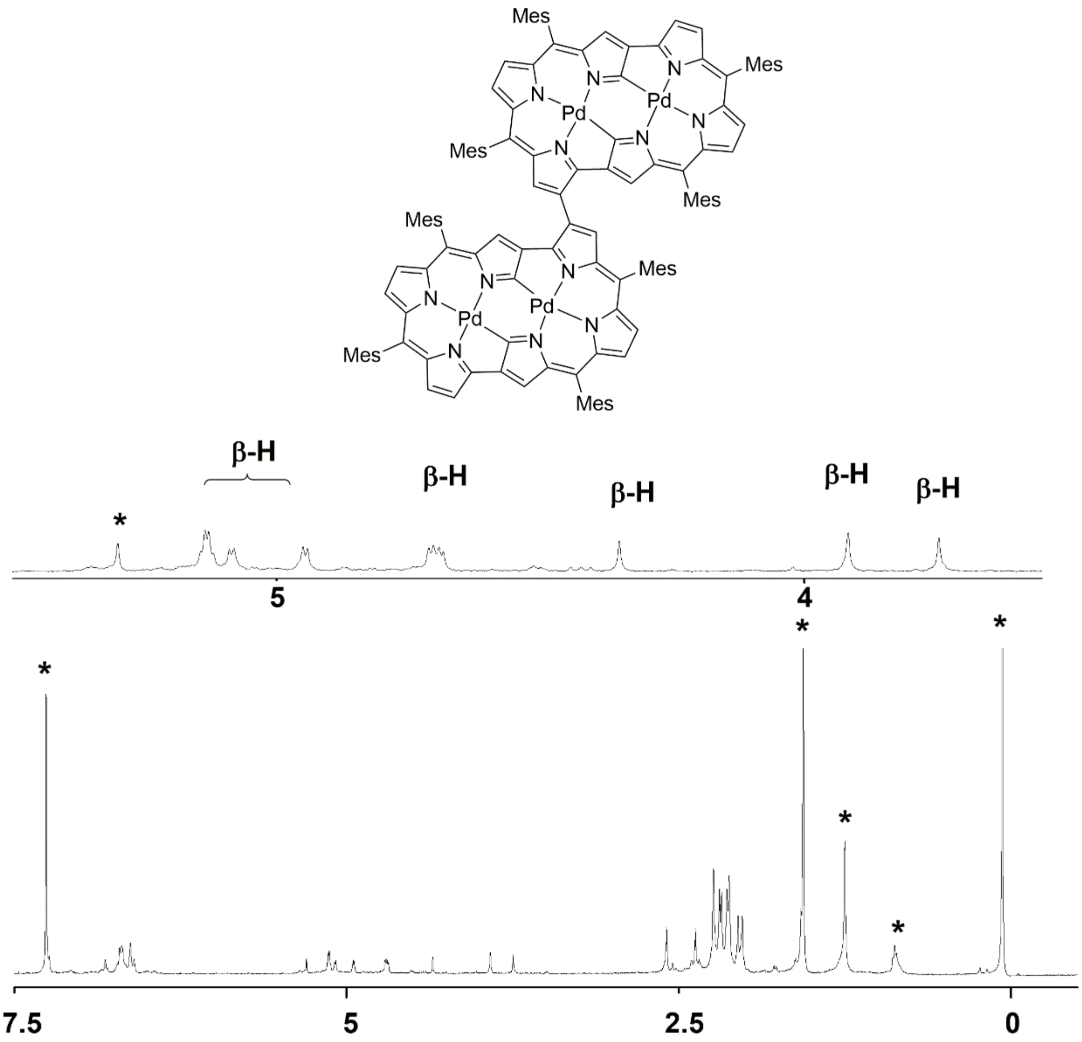

Supplementary Figure 20. **^{1}H NMR spectrum of 10 in $CDCl_3$.** *Solvent or impurities.

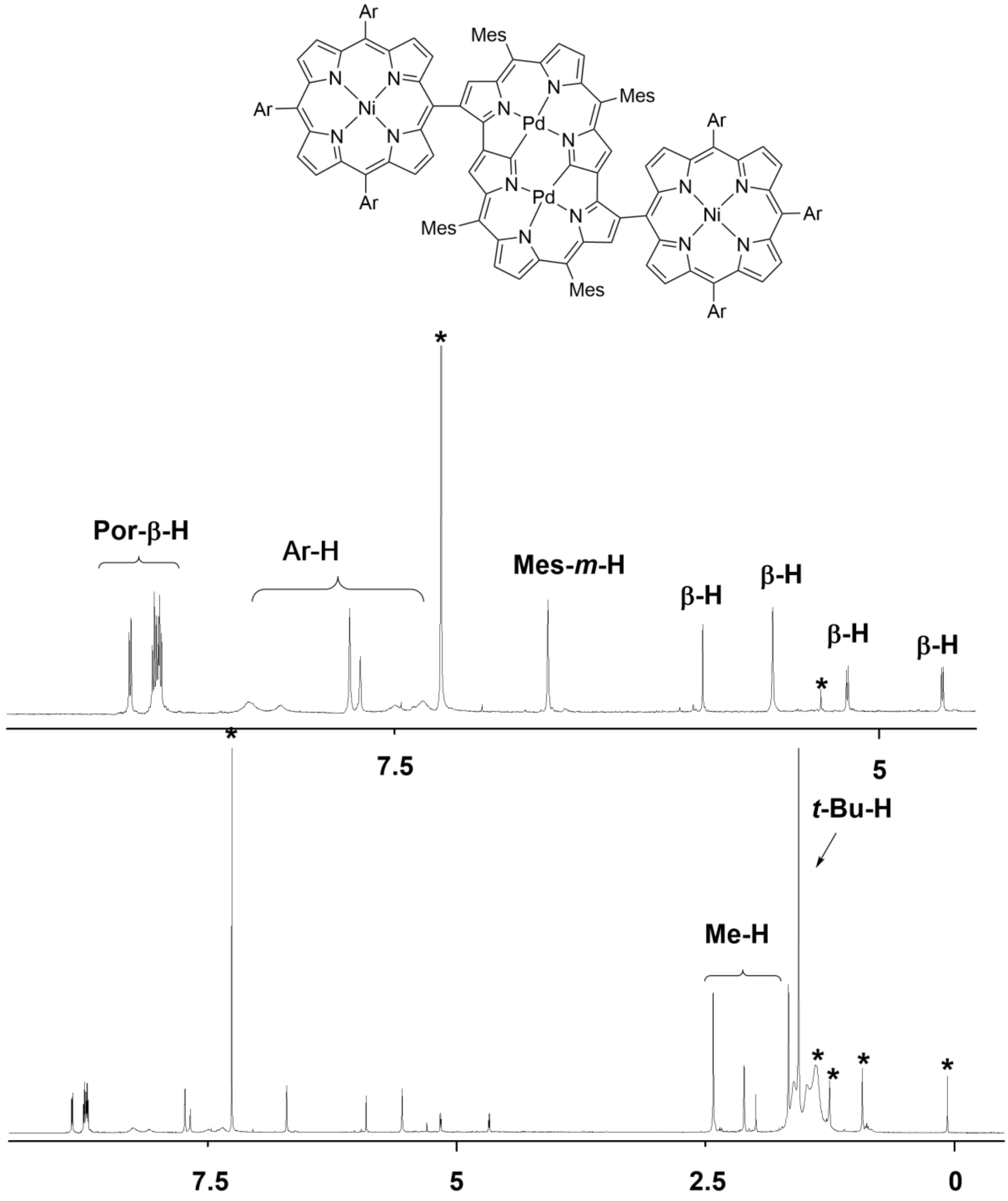

Supplementary Figure 21. **^{1}H NMR spectrum of 12 in $CDCl_3$.** *Solvent or impurities.

UV/Vis Absorption Spectra

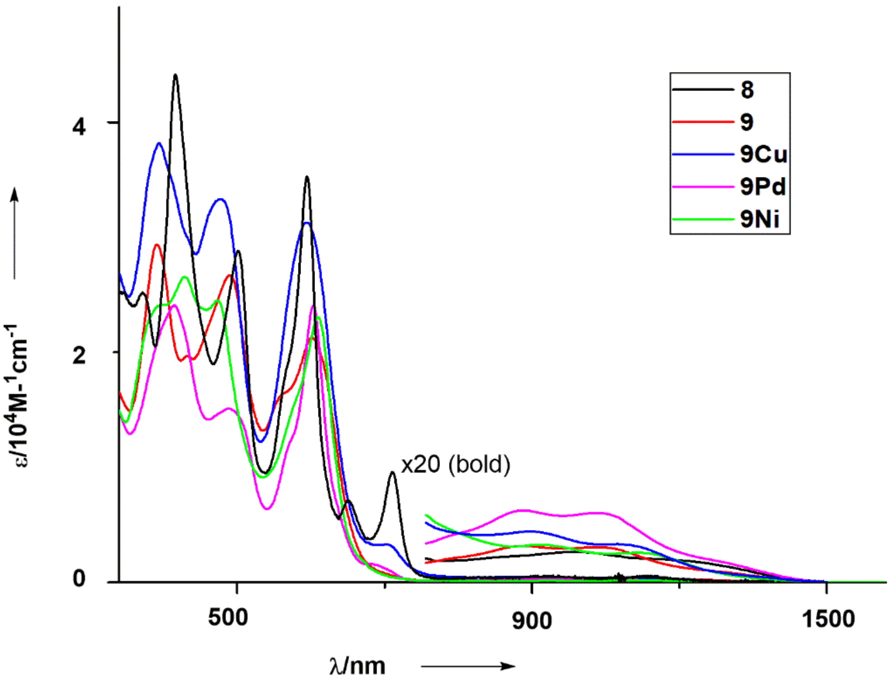

Supplementary Figure 22. **UV-Vis absorption spectra of 8, 9, 9Pd, 9Ni and 9Cu.** Black line, absorption spectrum of **8**; red line, absorption spectrum of **9**; blue line, absorption spectrum of **9Cu**; purple line, absorption spectrum of **9Pd**; green line, absorption spectrum of **9Ni**.

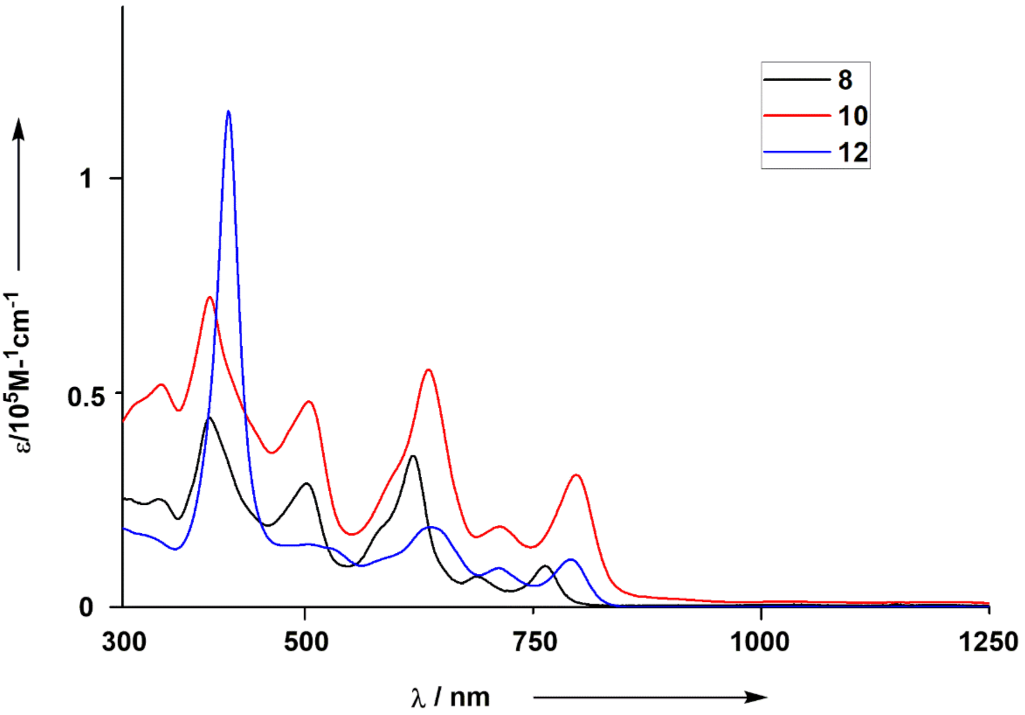

Supplementary Figure 23. **UV-Vis absorption spectra of 8, 10 and 12.** Black line, absorption

spectrum of **8**; red line, absorption spectrum of **10**; blue line, absorption spectrum of **12**.

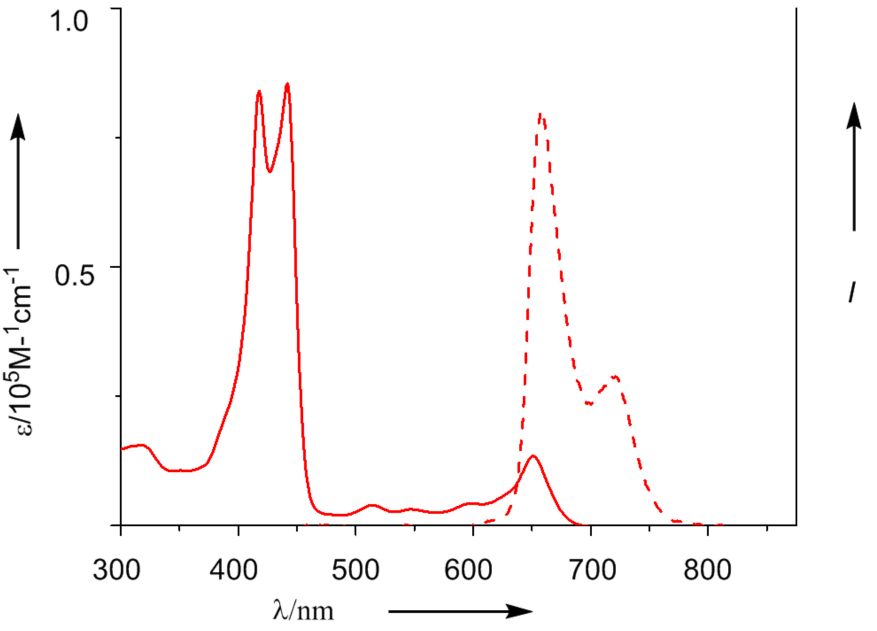

Supplementary Figure 24. **UV-Vis absorption spectrum and fluorescence spectrum of 6 in CH_2Cl_2.** Solid line, absorption spectrum; dash line, fluorescence spectrum.

MALDI-TOF-MS of Compounds

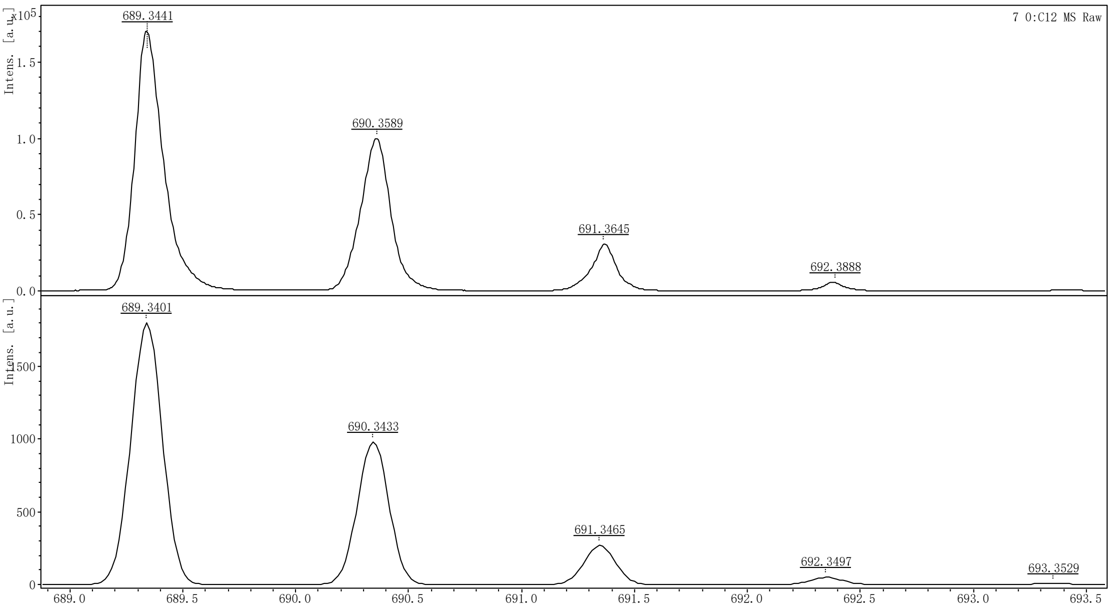

Supplementary Figure 25. **MALDI-TOF-MS spectrum of 6.** Top: observed; Bottom: simulated.

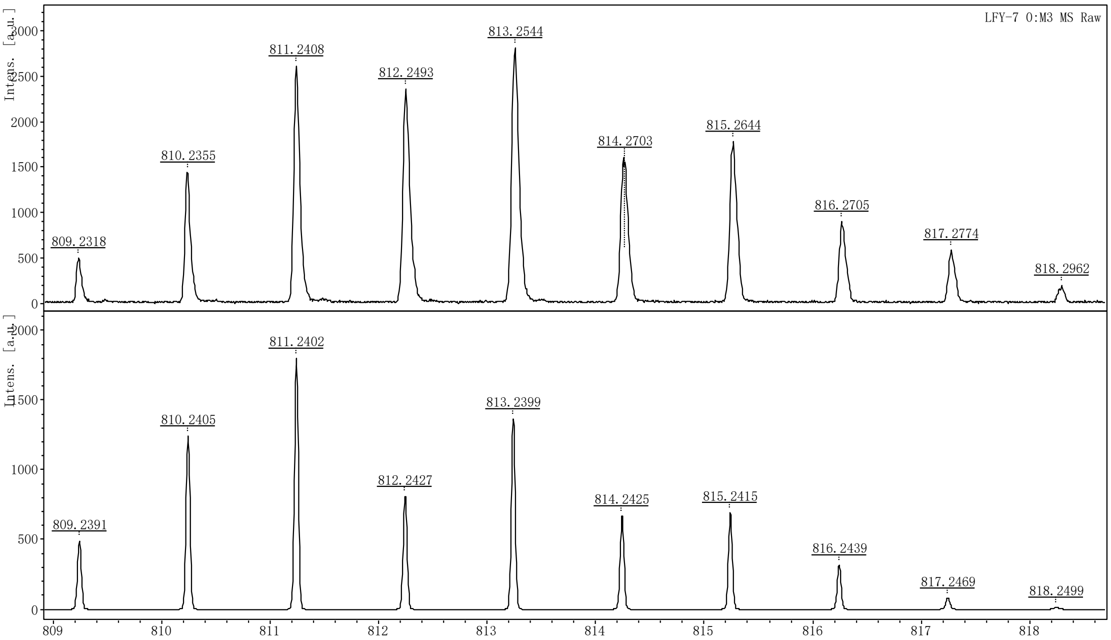

Supplementary Figure 26. **MALDI-TOF-MS spectrum of 7.** Top: observed; Bottom: simulated.

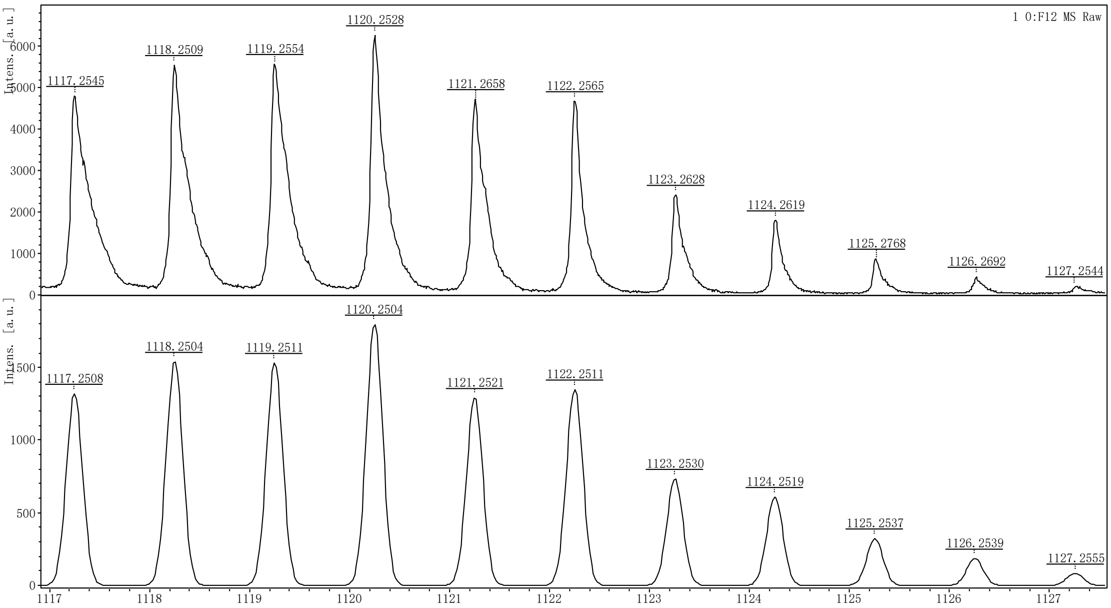

Supplementary Figure 27. **MALDI-TOF-MS spectrum of 8.** Top: observed; Bottom: simulated.

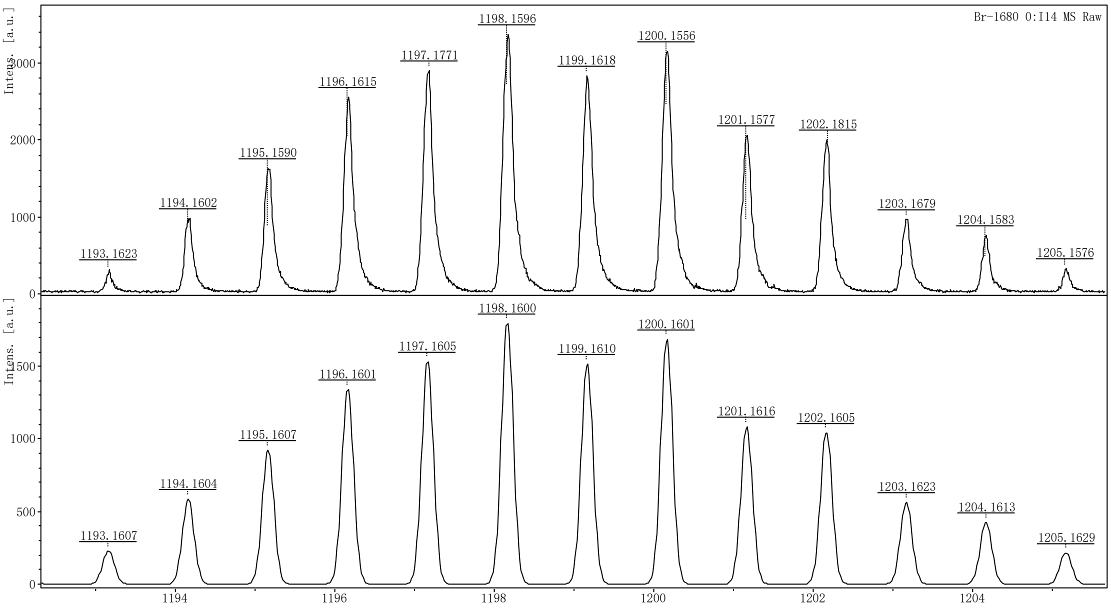

Supplementary Figure 28. **MALDI-TOF-MS spectrum of 8a.** Top: observed; Bottom: simulated.

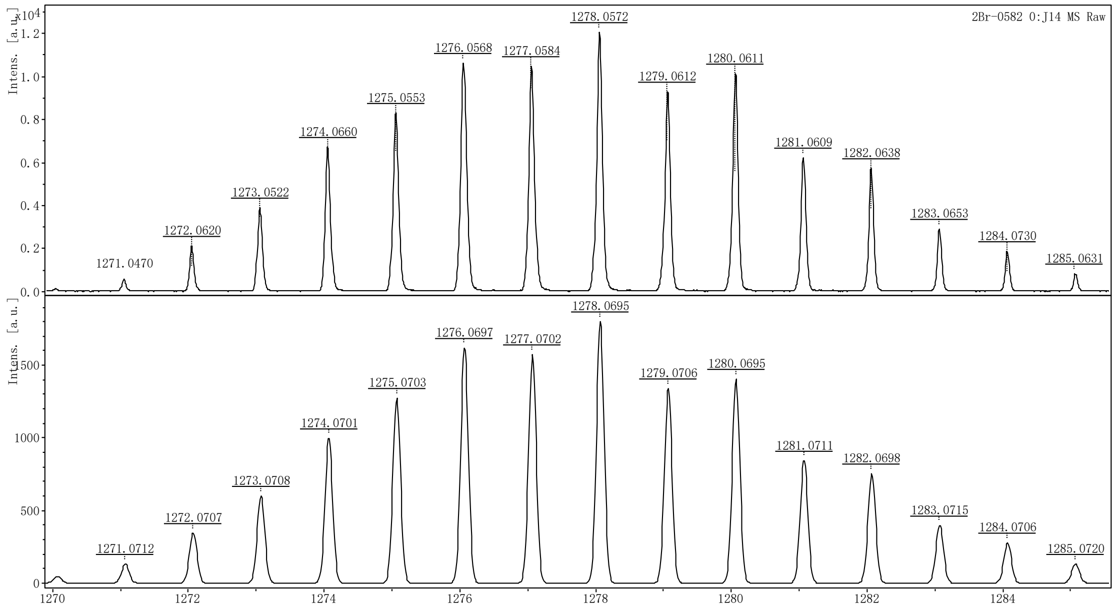

Supplementary Figure 29. MALDI-TOF-MS spectrum of 8b. Top: observed; Bottom: simulated.

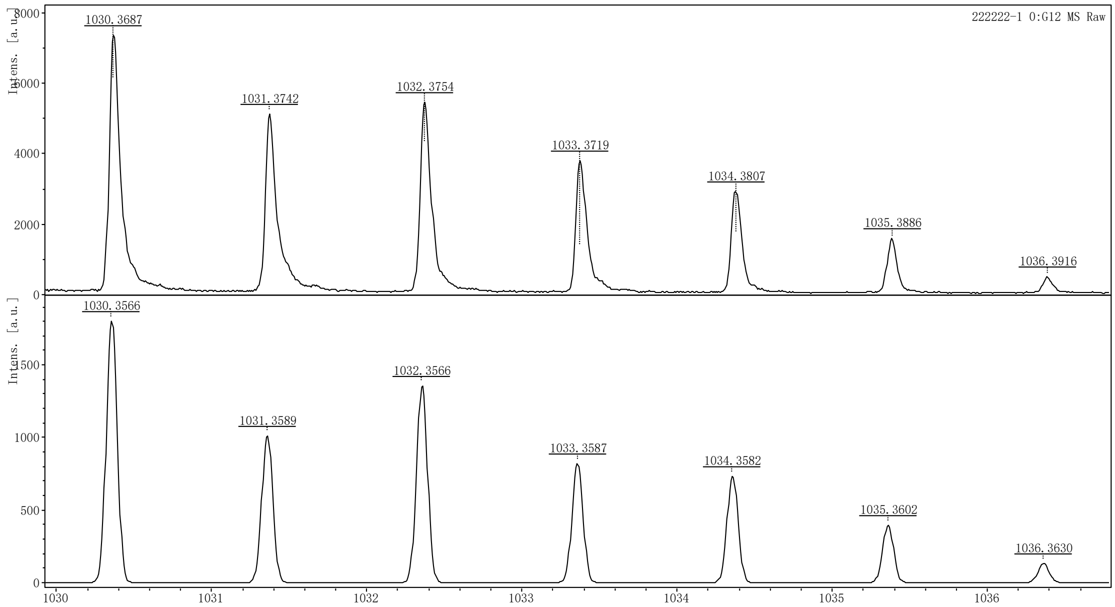

Supplementary Figure 30. **MALDI-TOF-MS spectrum of 9.** Top: observed; Bottom: simulated.

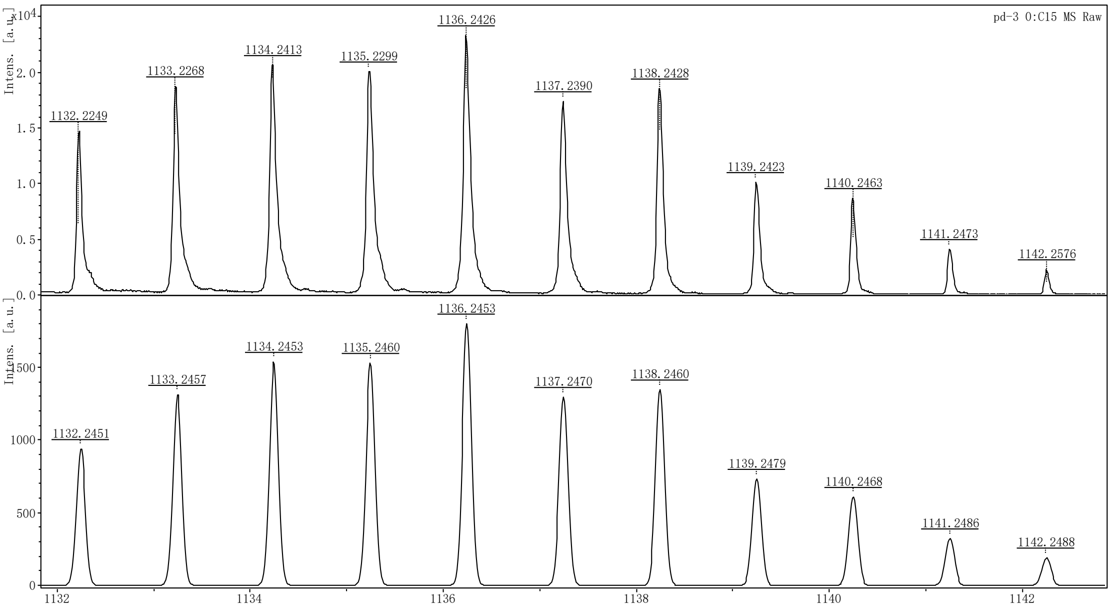

Supplementary Figure 31. **MALDI-TOF-MS spectrum of 9Pd.** Top: observed; Bottom: simulated.

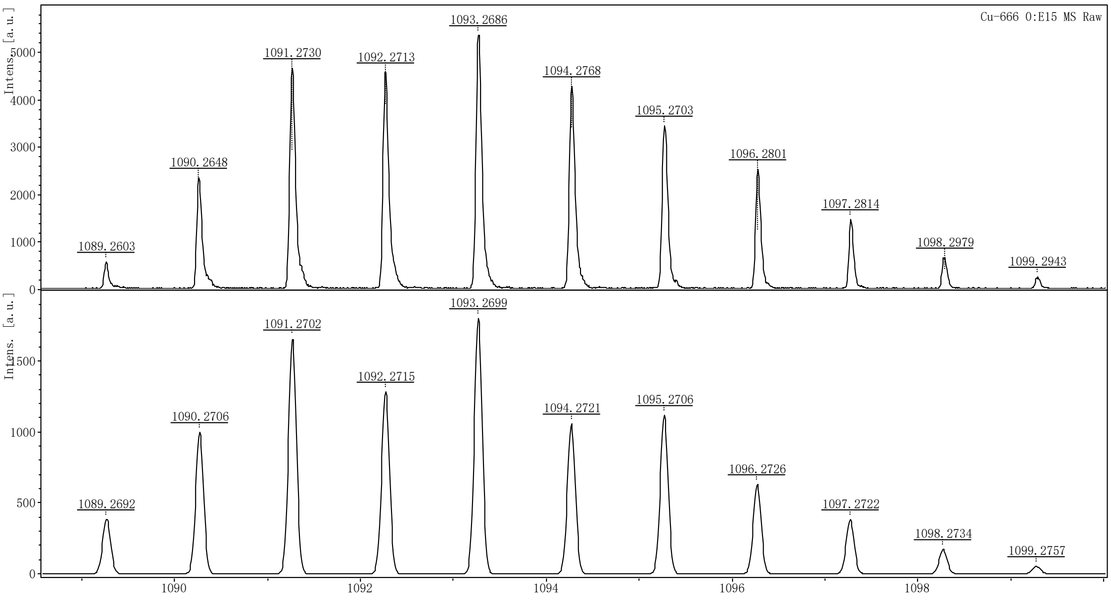

Supplementary Figure 32. **MALDI-TOF-MS spectrum of 9Cu.** Top: observed; Bottom: simulated.

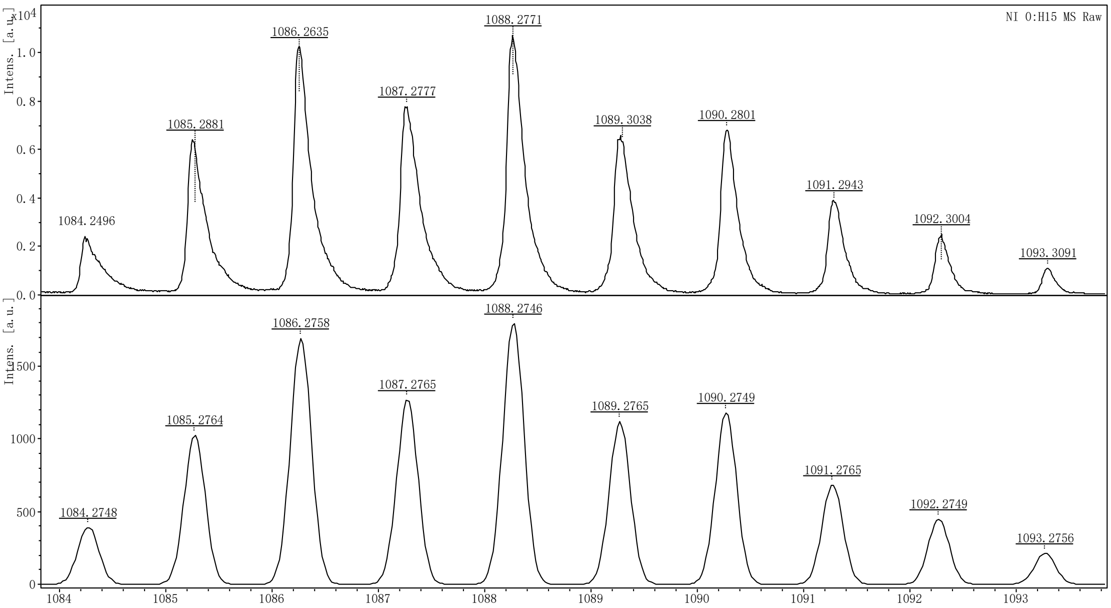

Supplementary Figure 33. **MALDI-TOF-MS spectrum of 9Ni.** Top: observed; Bottom: simulated.

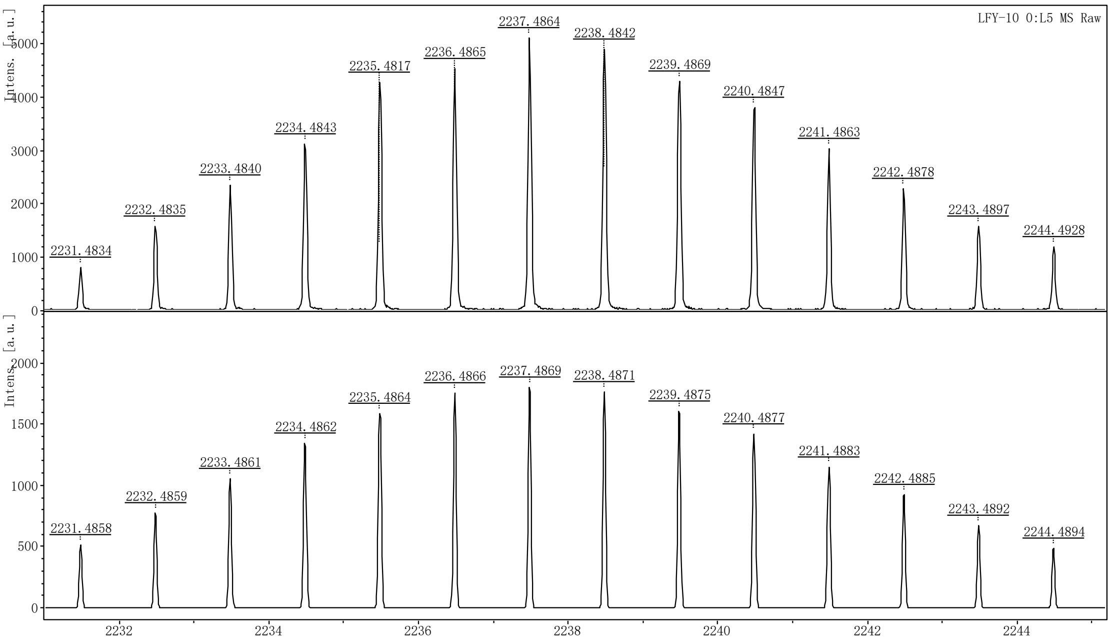

Supplementary Figure 34. **MALDI-TOF-MS spectrum of 10.** Top: observed; Bottom: simulated.

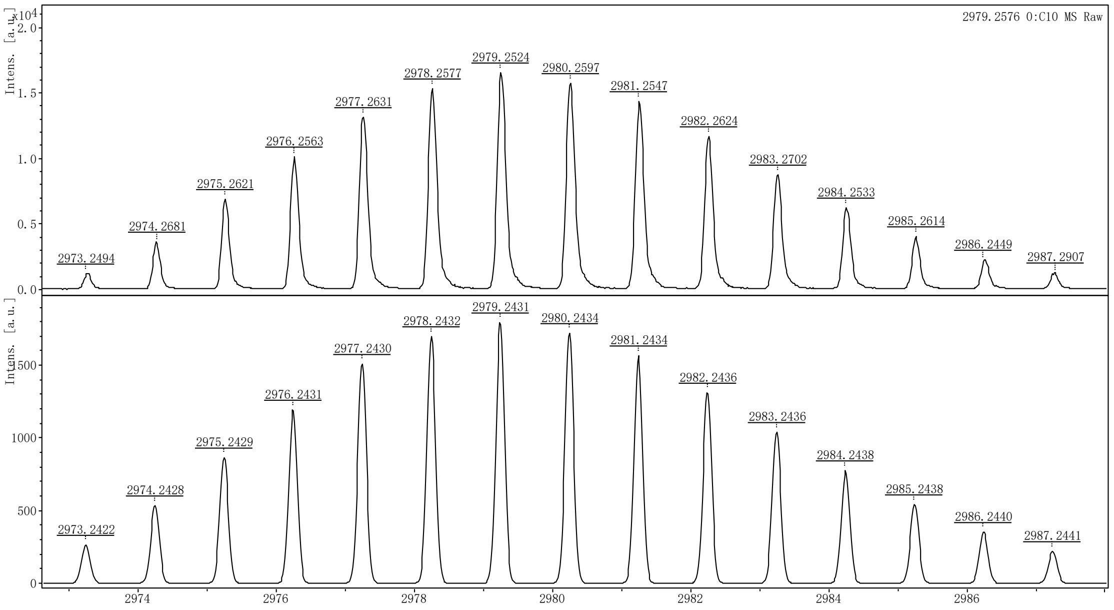

Supplementary Figure 35. **MALDI-TOF-MS spectrum of 12.** Top: observed; Bottom: simulated.

Electrochemical Data

Conditions: 0.1M $^{n}Bu_4NPF_6$ as electrolyte. Working electrode: Pt electrode, Counter electrode: Pt wire. Reference electrode: Ag/$AgNO_3$. Scan rate: 0.05 V/s. Potentials (V) vs. ferrocene/ferrocenium ion couple.

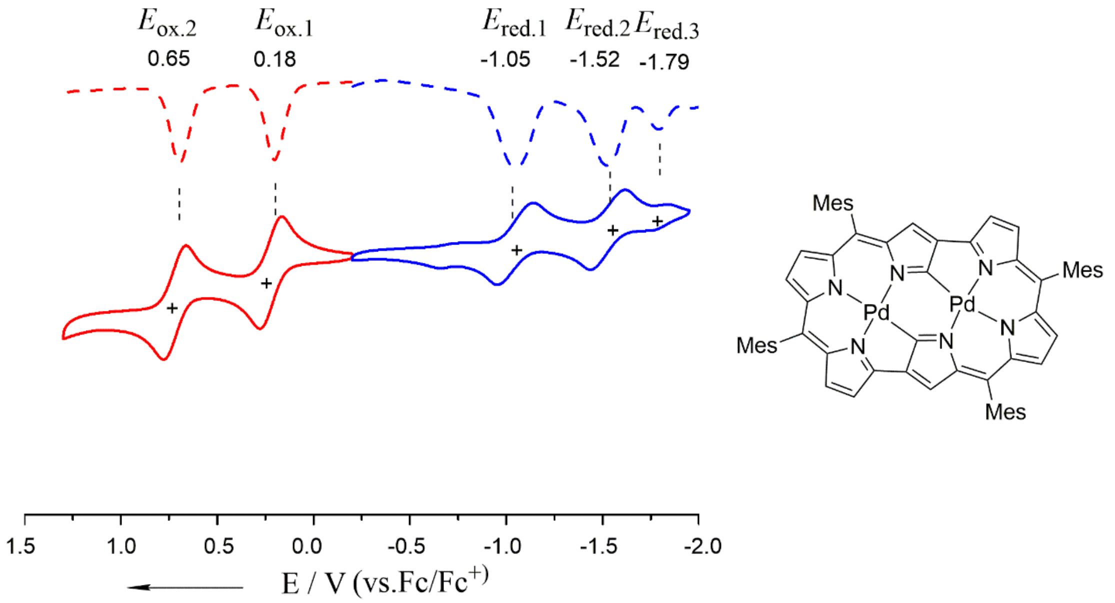

Supplementary Figure 36. **Cyclic voltammogram and differential-pulse voltammogram of 8.** Red line: oxidation potential; Blue line: reduction potential.

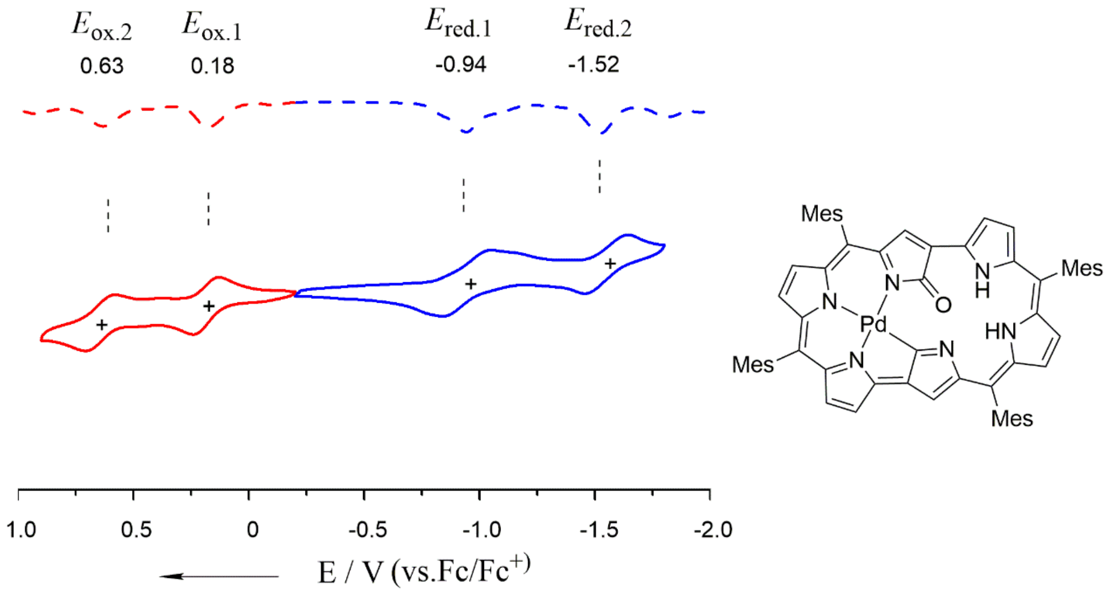

Supplementary Figure 37. Cyclic voltammogram and differential-pulse voltammogram of 9. Red line: oxidation potential; Blue line: reduction potential.

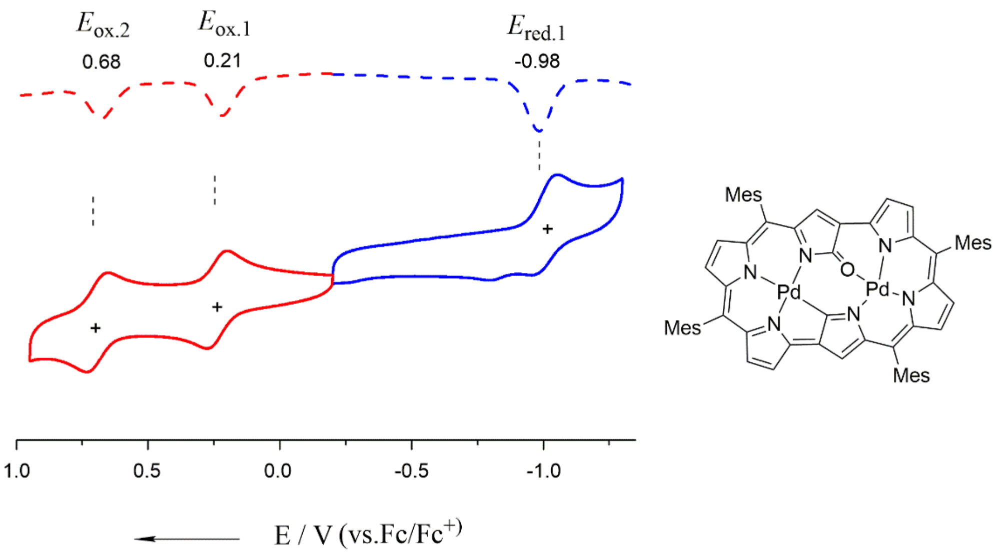

Supplementary Figure 38. Cyclic voltammogram and differential-pulse voltammogram of 9Pd. Red line: oxidation potential; Blue line: reduction potential.

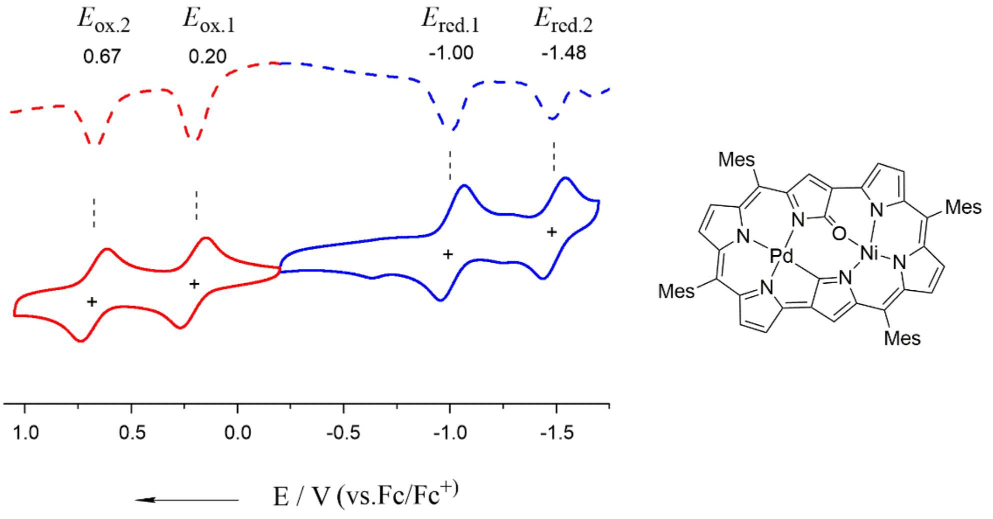

Supplementary Figure 39. Cyclic voltammogram and differential-pulse voltammogram of 9Ni.
Red line: oxidation potential; Blue line: reduction potential.

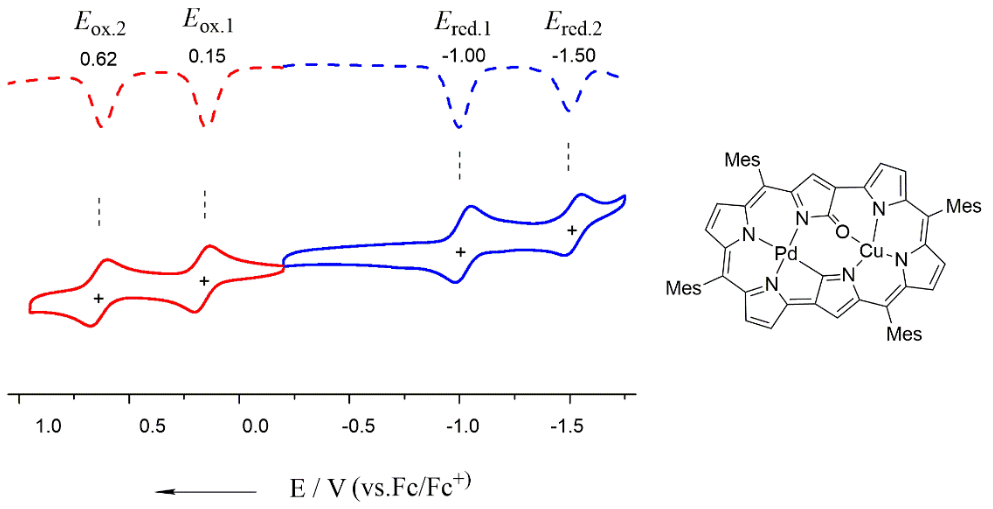

Supplementary Figure 40. Cyclic voltammogram and differential-pulse voltammogram of 9Cu.
Red line: oxidation potential; Blue line: reduction potential.

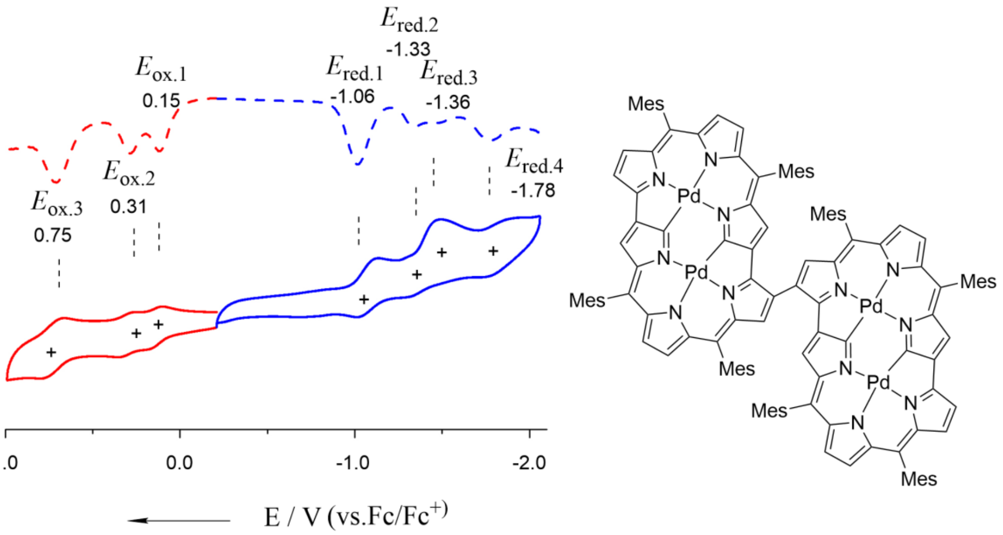

Supplementary Figure 41. Cyclic voltammogram and differential-pulse voltammogram of 10. Red line: oxidation potential; Blue line: reduction potential.

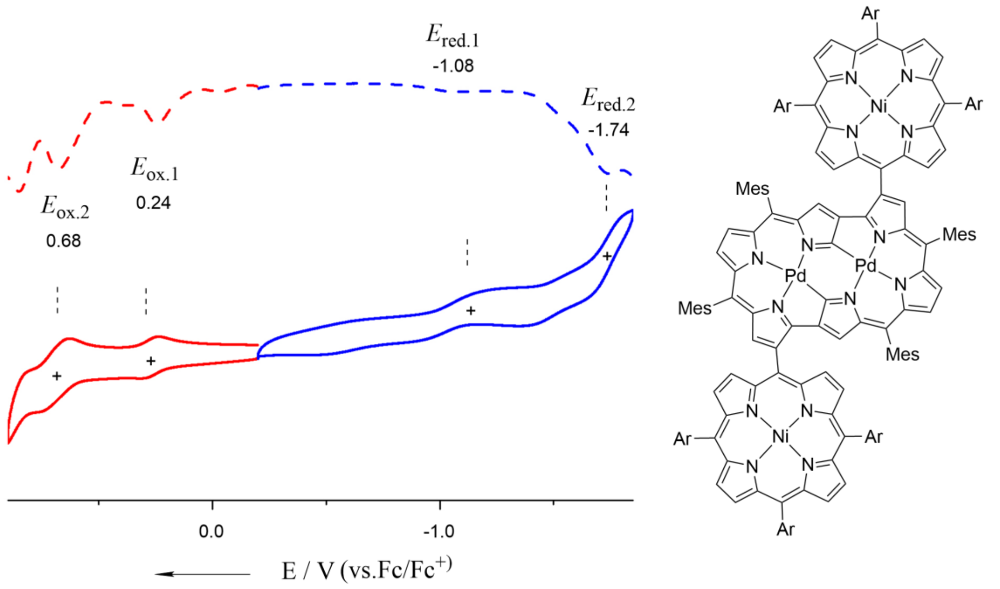

Supplementary Figure 42. Cyclic voltammogram and differential-pulse voltammogram of 12. Red line: oxidation potential; Blue line: reduction potential.

Supplementary Table 1. Redox potentials and electrochemical HOMO–LUMO gaps

sample	E_{Ox3} [V]	E_{Ox2} [V]	E_{Ox1} [V]	E_{Red1} [V]	E_{Red2} [V]	E_{Red3} [V]	E_{Red3} [V]	ΔE [eV][a]
**8**		0.65	0.18	-1.05	-1.52	-1.79		1.23
**9**		0.63	0.18	-0.94	-1.52			1.12
**9Pd**		0.68	0.21	-0.98				1.19
**9Ni**		0.67	0.20	-1.00	-1.48			1.20
**9Cu**		0.62	0.15	-1.00	-1.50			1.15

**10**	0.75	0.31	0.15	-1.06	-1.33	-1.36	-1.78	1.21
**12**		0.68	0.24	-1.08	-1.74			1.32

[a] ΔE = e(E_{ox1}-E_{red1}), that is, electrochemical HOMO–LUMO gaps.

X-Ray Crystal Data

Supplementary Table 2. Crystal data and refinement results for compound **6**.

Identification code	exp_1242	
Empirical formula	$C_{49}H_{43}N_3O$	
Formula weight	689.43	
Temperature	100.01(10) K	
Wavelength	1.54184 Å	
Crystal system	Monoclinic	
Space group	C 1 c 1	
Unit cell dimensions	a = 19.2386(5) Å	α= 90°.
	b = 14.0313(3) Å	β= 95.628(2)°.
	c = 15.2313(4) Å	γ = 90°.
Volume	4091.76(18) $Å^3$	
Z	4	
Density (calculated)	1.314 Mg/m^3	
Absorption coefficient	2.353 mm^{-1}	
F(000)	1696	
Crystal size	0.1 x 0.1 x 0.05 mm^3	
Theta range for data collection	3.906 to 66.595°.	
Index ranges	-21<=h<=22, -16<=k<=16, -16<=l<=18	
Reflections collected	7244	
Independent reflections	4376 [R(int) = 0.0351]	
Completeness to theta = 66.595°	100.0 %	
Absorption correction	Semi-empirical from equivalents	
Max. and min. transmission	1.00000 and 0.75763	
Refinement method	Full-matrix least-squares on F^2	
Data / restraints / parameters	4376 / 131 / 520	
Goodness-of-fit on F^2	1.021	
Final R indices [I>2sigma(I)]	R1 = 0.0734, wR2 = 0.1932	
R indices (all data)	R1 = 0.0759, wR2 = 0.1978	
Absolute structure parameter	0.03(3)	
Extinction coefficient	n/a	
Largest diff. peak and hole	0.813 and -0.879 e.$Å^{-3}$	
CCDC	2239867	

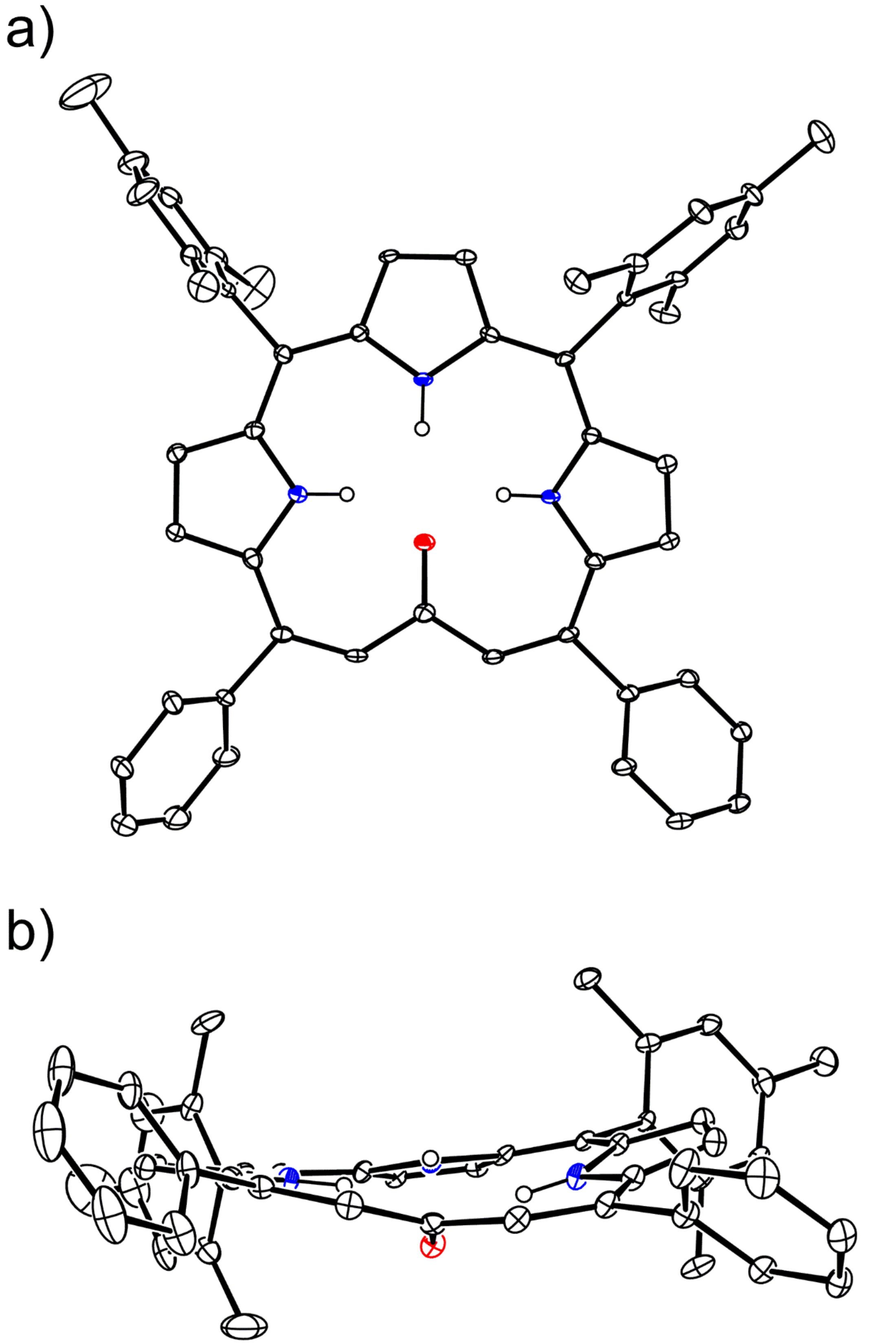

Supplementary Figure 43. X-ray crystal structure of 6. (a) Top view and (b) side view. The thermal ellipsoids are drawn at 30% probability level.

Supplementary Table 3. Crystal data and refinement results for compound **7**.

Identification code	exp_1658	
Empirical formula	$C_{49}H_{43}N_3O_2Pd$	
Formula weight	812.24	
Temperature	99.9(2) K	
Wavelength	1.54184 Å	
Crystal system	Orthorhombic	
Space group	Iba2	
Unit cell dimensions	a = 34.2146(5) Å	α= 90°.
	b = 15.9294(2) Å	β= 90°.
	c = 14.1391(2) Å	γ = 90°.
Volume	7706.06(18) $Å^3$	
Z	8	
Density (calculated)	1.400 Mg/m^3	
Absorption coefficient	4.232 mm^{-1}	
F(000)	3360	
Crystal size	0.1 x 0.1 x 0.1 mm^3	
Theta range for data collection	2.583 to 66.568°.	
Index ranges	-40<=h<=40, -18<=k<=18, -16<=l<=11	
Reflections collected	24293	
Independent reflections	5342 [R(int) = 0.0311]	
Completeness to theta = 66.568°	100.0 %	
Absorption correction	Semi-empirical from equivalents	
Max. and min. transmission	1.00000 and 0.67272	
Refinement method	Full-matrix least-squares on F^2	
Data / restraints / parameters	5342 / 1 / 503	
Goodness-of-fit on F^2	1.038	
Final R indices [I>2sigma(I)]	R1 = 0.0233, wR2 = 0.0576	
R indices (all data)	R1 = 0.0256, wR2 = 0.0586	
Absolute structure parameter	-0.029(5)	
Extinction coefficient	n/a	
Largest diff. peak and hole	0.307 and -0.468 e.$Å^{-3}$	
CCDC	2239868	

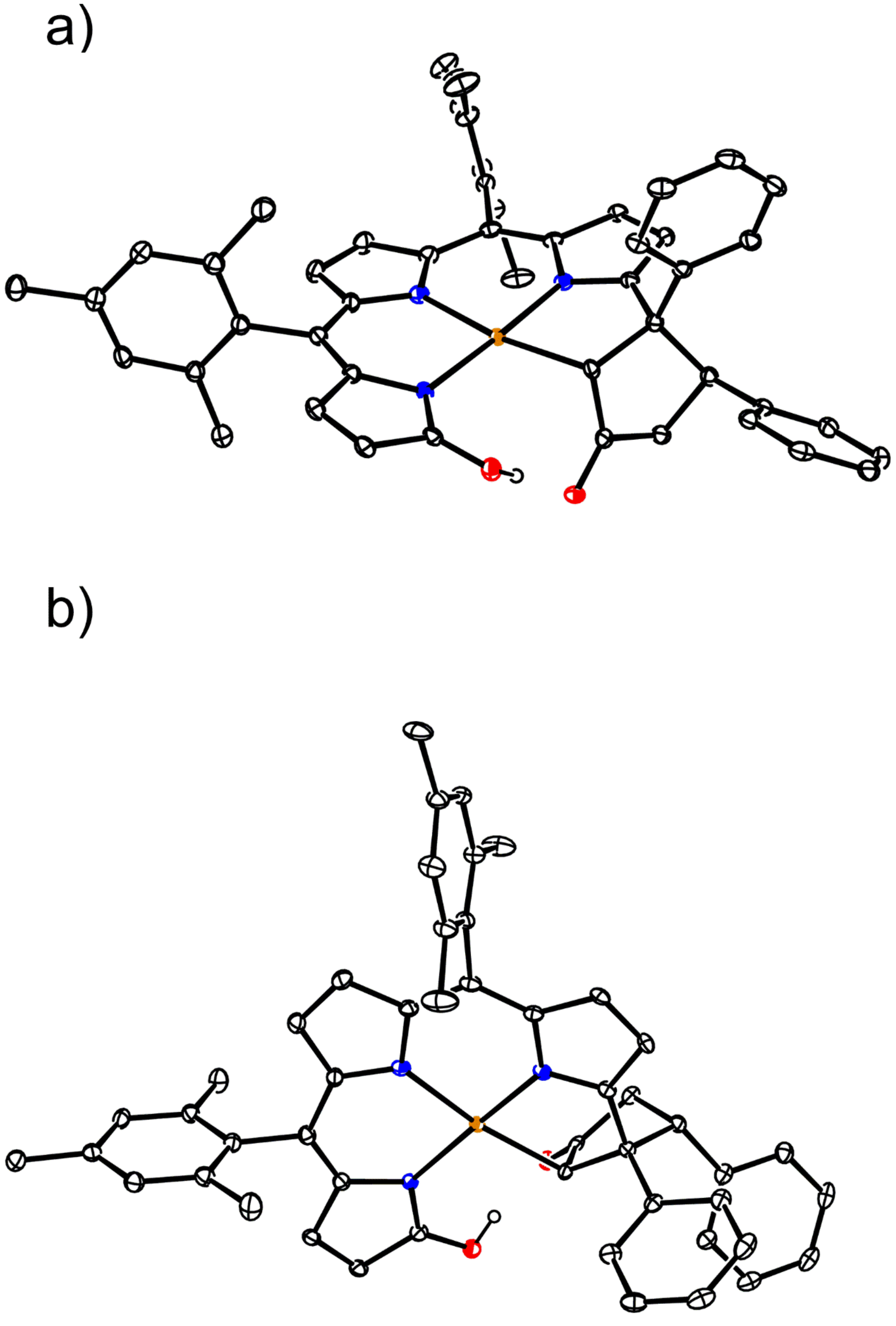

Supplementary Figure 44. X-ray crystal structure of 7. (a) Top view and (b) side view. The thermal ellipsoids are drawn at 30% probability level.

Supplementary Table 4. Crystal data and refinement results for compound **8**.

Identification code	exp_1335	
Empirical formula	$C_{64}H_{54}N_6Pd_2$	
Formula weight	1120.25	
Temperature	100.01(10) K	
Wavelength	1.54184 Å	
Crystal system	Monoclinic	
Space group	P 1 21/c 1	
Unit cell dimensions	a = 15.5926(4) Å	α= 90°.
	b = 11.3061(3) Å	β= 104.232(3)°.
	c = 16.9999(5) Å	γ = 90°.
Volume	2904.95(15) Å^3	
Z	2	
Density (calculated)	1.553 Mg/m^3	
Absorption coefficient	7.907 mm^{-1}	
F(000)	1376	
Crystal size	0.1 x 0.1 x 0.02 mm^3	
Theta range for data collection	2.924 to 66.596°.	
Index ranges	-18<=h<=18, -13<=k<=12, -20<=l<=9	
Reflections collected	10579	
Independent reflections	5136 [R(int) = 0.0496]	
Completeness to theta = 66.596°	99.9 %	
Absorption correction	Semi-empirical from equivalents	
Max. and min. transmission	1.00000 and 0.65999	
Refinement method	Full-matrix least-squares on F^2	
Data / restraints / parameters	5136 / 0 / 367	
Goodness-of-fit on F^2	1.028	
Final R indices [I>2sigma(I)]	R1 = 0.0513, wR2 = 0.1299	
R indices (all data)	R1 = 0.0639, wR2 = 0.1406	
Extinction coefficient	n/a	
Largest diff. peak and hole	1.904 and -1.056 e.Å^{-3}	
CCDC	2239869	

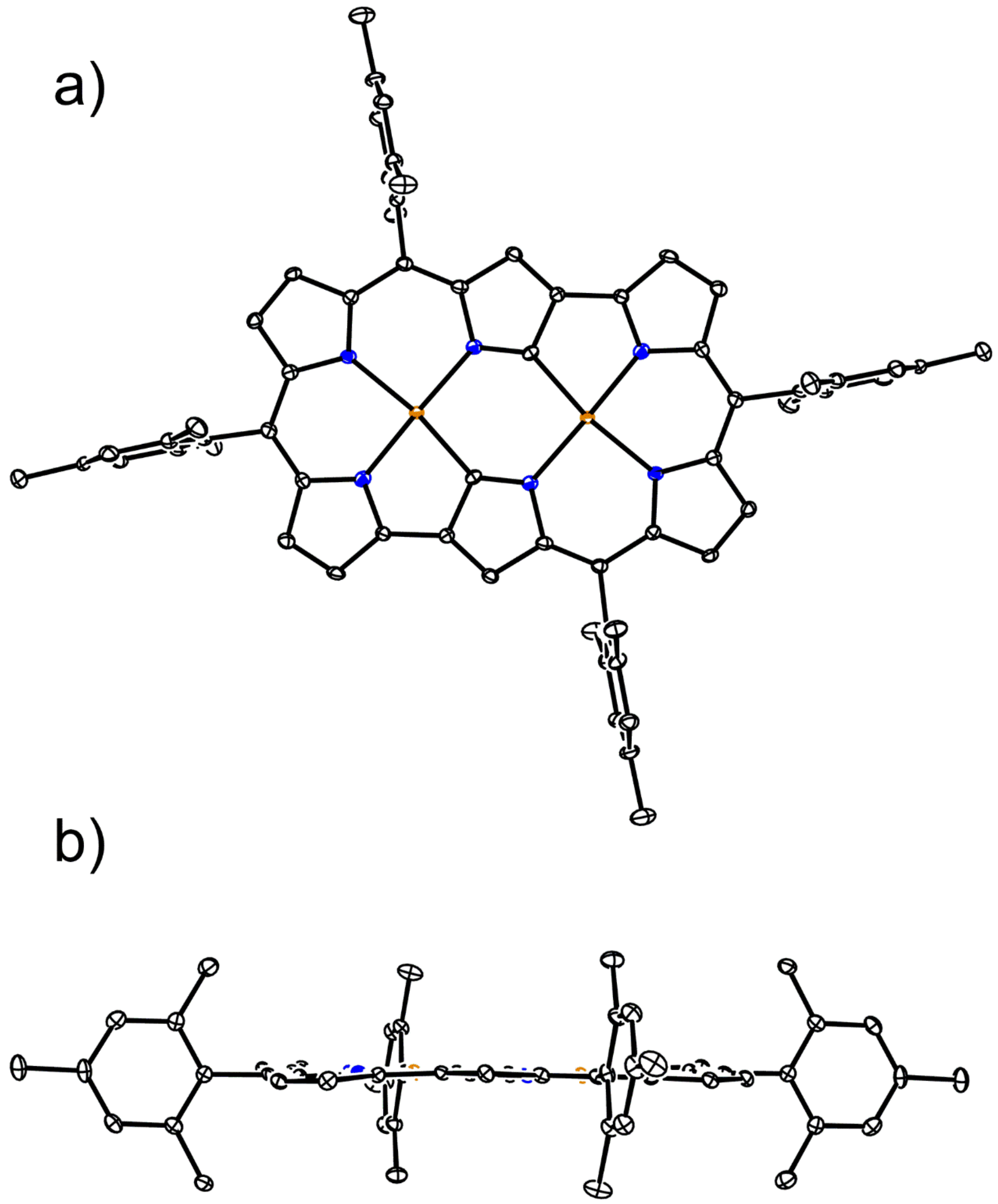

Supplementary Figure 45. X-ray crystal structure of 8. (a) Top view and (b) side view. The thermal ellipsoids are drawn at 30% probability level.

Supplementary Table 5. Crystal data and refinement results for compound **8a**.

Identification code	exp_2204_sq	
Empirical formula	C64 H53 Br N6 Pd2	
Formula weight	1198.83	
Temperature	100.01(10) K	
Wavelength	1.54184 Å	
Crystal system	Monoclinic	
Space group	P 1 21/c 1	
Unit cell dimensions	a = 15.7666(5) Å	α= 90°.
	b = 13.1945(3) Å	β= 114.654(3)°.
	c = 15.9606(5) Å	γ = 90°.
Volume	3017.65(16) Å^3	
Z	2	
Density (calculated)	1.319 Mg/m^3	
Absorption coefficient	5.900 mm^{-1}	
F(000)	1212	
Crystal size	0.2 x 0.1 x 0.1 mm^3	
Theta range for data collection	3.084 to 66.599°.	
Index ranges	-17<=h<=18, -14<=k<=15, -18<=l<=16	
Reflections collected	10417	
Independent reflections	5344 [R(int) = 0.0434]	
Completeness to theta = 66.599°	100.0 %	
Absorption correction	Semi-empirical from equivalents	
Max. and min. transmission	1.00000 and 0.34266	
Refinement method	Full-matrix least-squares on F^2	
Data / restraints / parameters	5344 / 6 / 340	
Goodness-of-fit on F^2	1.079	
Final R indices [I>2sigma(I)]	R1 = 0.0516, wR2 = 0.1434	
R indices (all data)	R1 = 0.0591, wR2 = 0.1513	
Extinction coefficient	n/a	
Largest diff. peak and hole	1.119 and -0.889 e.Å^{-3}	
CCDC	2239872	

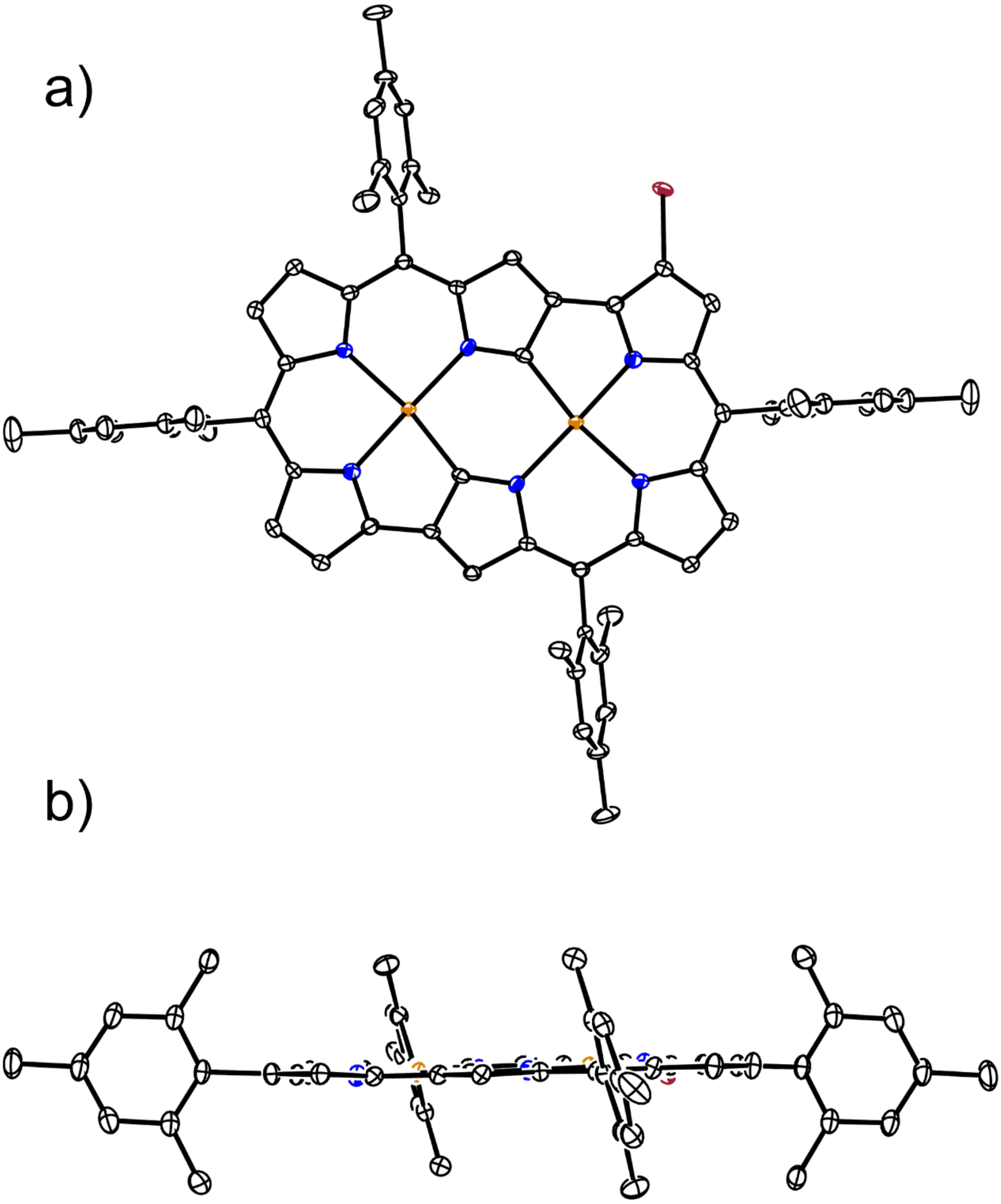

Supplementary Figure 46. X-ray crystal structure of 8a. (a) Top view and (b) side view. The thermal ellipsoids are drawn at 30% probability level.

Supplementary Table 6. Crystal data and refinement results for compound **8b**.

Identification code	exp_2202	
Empirical formula	$C_{64}H_{52}Br_2N_6Pd_2$	
Formula weight	1278.07	
Temperature	100.01(10) K	
Wavelength	1.54184 Å	
Crystal system	Monoclinic	
Space group	P 1 21/c 1	
Unit cell dimensions	a = 15.5587(8) Å	α= 90°.
	b = 13.3543(5) Å	β= 113.942(6)°.
	c = 15.9419(8) Å	γ = 90°.
Volume	3027.3(3) $Å^3$	
Z	2	
Density (calculated)	1.664 Mg/m^3	
Absorption coefficient	9.146 mm^{-1}	
F(000)	1512	
Crystal size	0.3 x 0.2 x 0.1 mm^3	
Theta range for data collection	3.108 to 66.589°.	
Index ranges	-12<=h<=18, -15<=k<=9, -18<=l<=18	
Reflections collected	9953	
Independent reflections	5338 [R(int) = 0.0424]	
Completeness to theta = 66.589°	99.9 %	
Absorption correction	Semi-empirical from equivalents	
Max. and min. transmission	1.00000 and 0.11955	
Refinement method	Full-matrix least-squares on F^2	
Data / restraints / parameters	5338 / 0 / 386	
Goodness-of-fit on F^2	1.013	
Final R indices [I>2sigma(I)]	R1 = 0.0648, wR2 = 0.1691	
R indices (all data)	R1 = 0.0695, wR2 = 0.1751	
Extinction coefficient	n/a	
Largest diff. peak and hole	2.452 and -1.613 e.$Å^{-3}$	
CCDC	2239870	

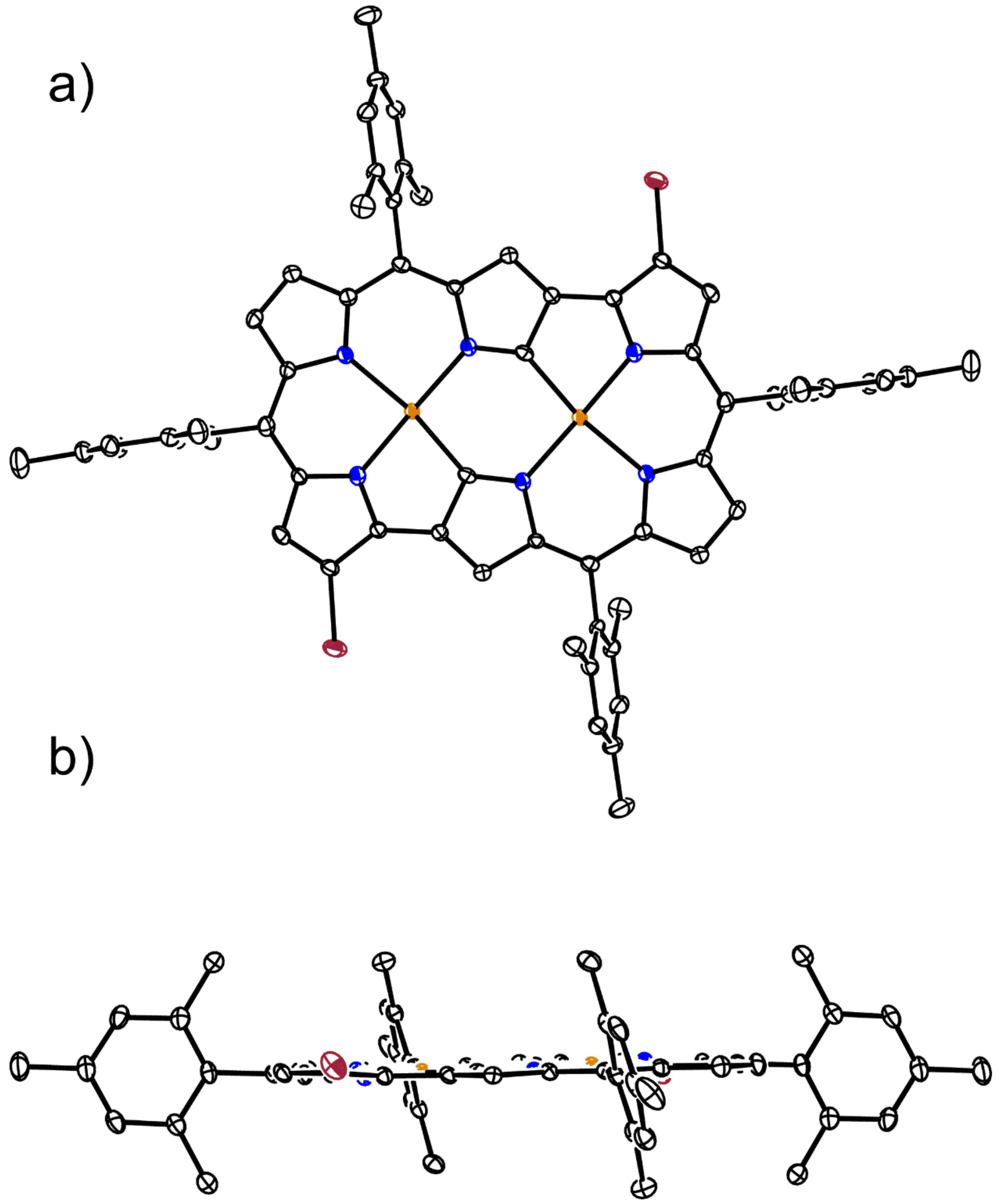

Supplementary Figure 47. X-ray crystal structure of 8b. (a) Top view and (b) side view. The thermal ellipsoids are drawn at 30% probability level.

Supplementary Table 7. Crystal data and refinement results for compound **9**.

Identification code	exp_2188_sq_sq	
Empirical formula	C64 H56 N6 O Pd	
Formula weight	1031.54	
Temperature	100.01(16) K	
Wavelength	1.54184 Å	
Crystal system	Monoclinic	
Space group	P 1 21/c 1	
Unit cell dimensions	a = 15.8106(14) Å	α= 90°.
	b = 11.2102(10) Å	β= 105.385(11)°.
	c = 17.812(3) Å	γ = 90°.
Volume	3043.8(6) Å^3	
Z	2	
Density (calculated)	1.126 Mg/m^3	
Absorption coefficient	2.782 mm^{-1}	
F(000)	1072	
Crystal size	0.2 x 0.2 x 0.1 mm^3	
Theta range for data collection	2.899 to 66.587°.	
Index ranges	-18<=h<=15, -6<=k<=13, -16<=l<=21	
Reflections collected	10376	
Independent reflections	5374 [R(int) = 0.0436]	
Completeness to theta = 66.587°	99.9 %	
Absorption correction	Semi-empirical from equivalents	
Max. and min. transmission	1.00000 and 0.53974	
Refinement method	Full-matrix least-squares on F^2	
Data / restraints / parameters	5374 / 77 / 377	
Goodness-of-fit on F^2	1.054	
Final R indices [I>2sigma(I)]	R1 = 0.0898, wR2 = 0.2573	
R indices (all data)	R1 = 0.1190, wR2 = 0.2816	
Extinction coefficient	n/a	
Largest diff. peak and hole	0.338 and -0.462 e.Å^{-3}	
CCDC	2239871	

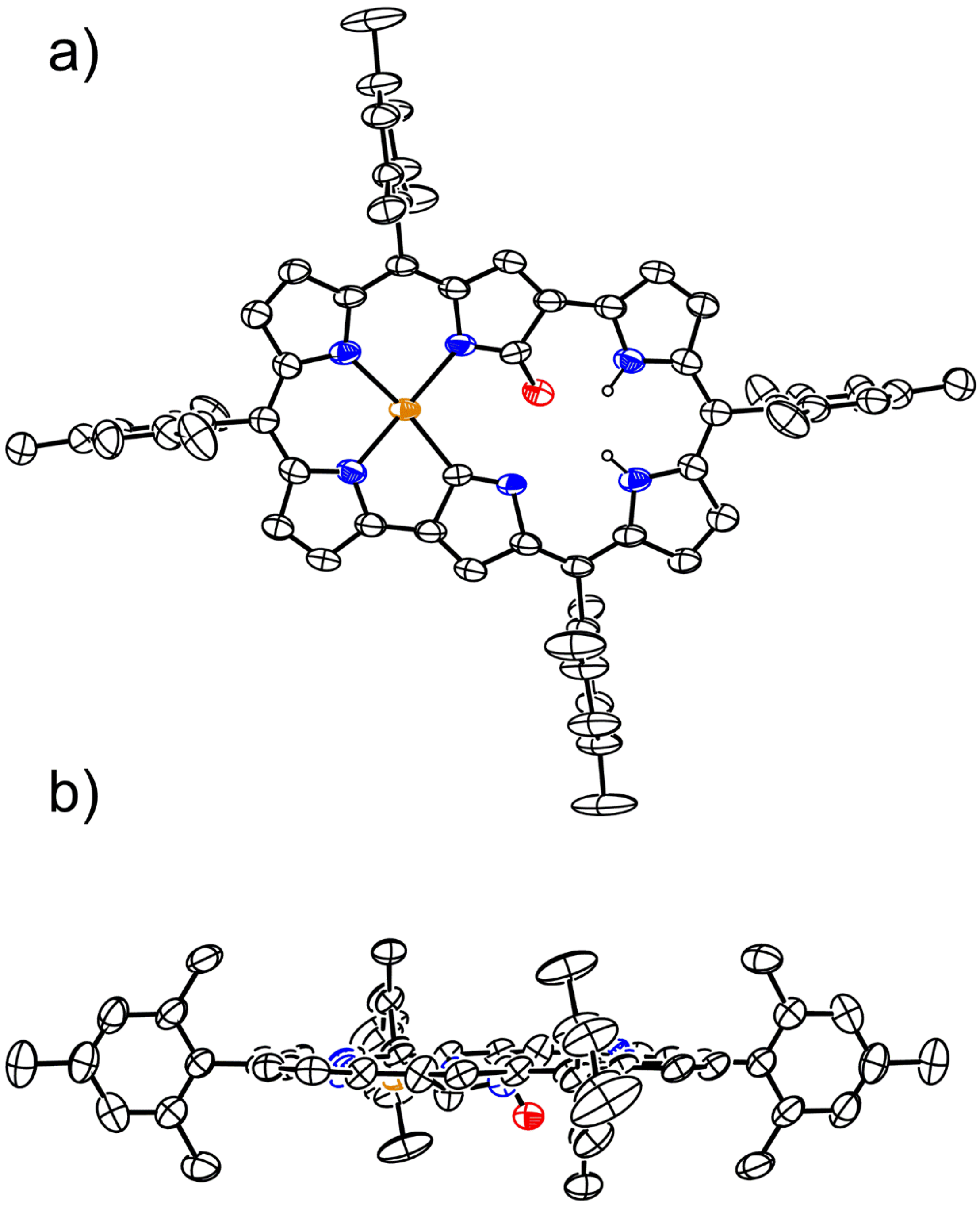

Supplementary Figure 48. X-ray crystal structure of 9. (a) Top view and (b) side view. The thermal ellipsoids are drawn at 30% probability level.

Supplementary Table 8. Crystal data and refinement results for compound **9Pd**.

Identification code	exp_2974_sq	
Empirical formula	C64 H54 N6 O Pd2	
Formula weight	1135.93	
Temperature	100.01(10) K	
Wavelength	1.54184 Å	
Crystal system	Triclinic	
Space group	P-1	
Unit cell dimensions	a = 16.6964(4) Å	α= 85.197(2)°.
	b = 19.3933(5) Å	β= 71.747(2)°.
	c = 20.8209(5) Å	γ = 68.099(2)°.
Volume	5936.3(3) $Å^3$	
Z	4	
Density (calculated)	1.271 Mg/m^3	
Absorption coefficient	5.224 mm^{-1}	
F(000)	2320	
Crystal size	0.5 x 0.5 x 0.3 mm^3	
Theta range for data collection	2.457 to 66.597°.	
Index ranges	-19<=h<=19, -22<=k<=23, -24<=l<=23	
Reflections collected	39568	
Independent reflections	20959 [R(int) = 0.0567]	
Completeness to theta = 66.597°	99.9 %	
Absorption correction	Semi-empirical from equivalents	
Max. and min. transmission	1.00000 and 0.26036	
Refinement method	Full-matrix least-squares on F2	
Data / restraints / parameters	20959 / 472 / 1459	
Goodness-of-fit on F^2	1.024	
Final R indices [I>2sigma(I)]	R1 = 0.0720, wR2 = 0.1857	
R indices (all data)	R1 = 0.0848, wR2 = 0.1999	
Extinction coefficient	n/a	
Largest diff. peak and hole	2.900 and -2.972 e.$Å^{-3}$	
CCDC	2278452	

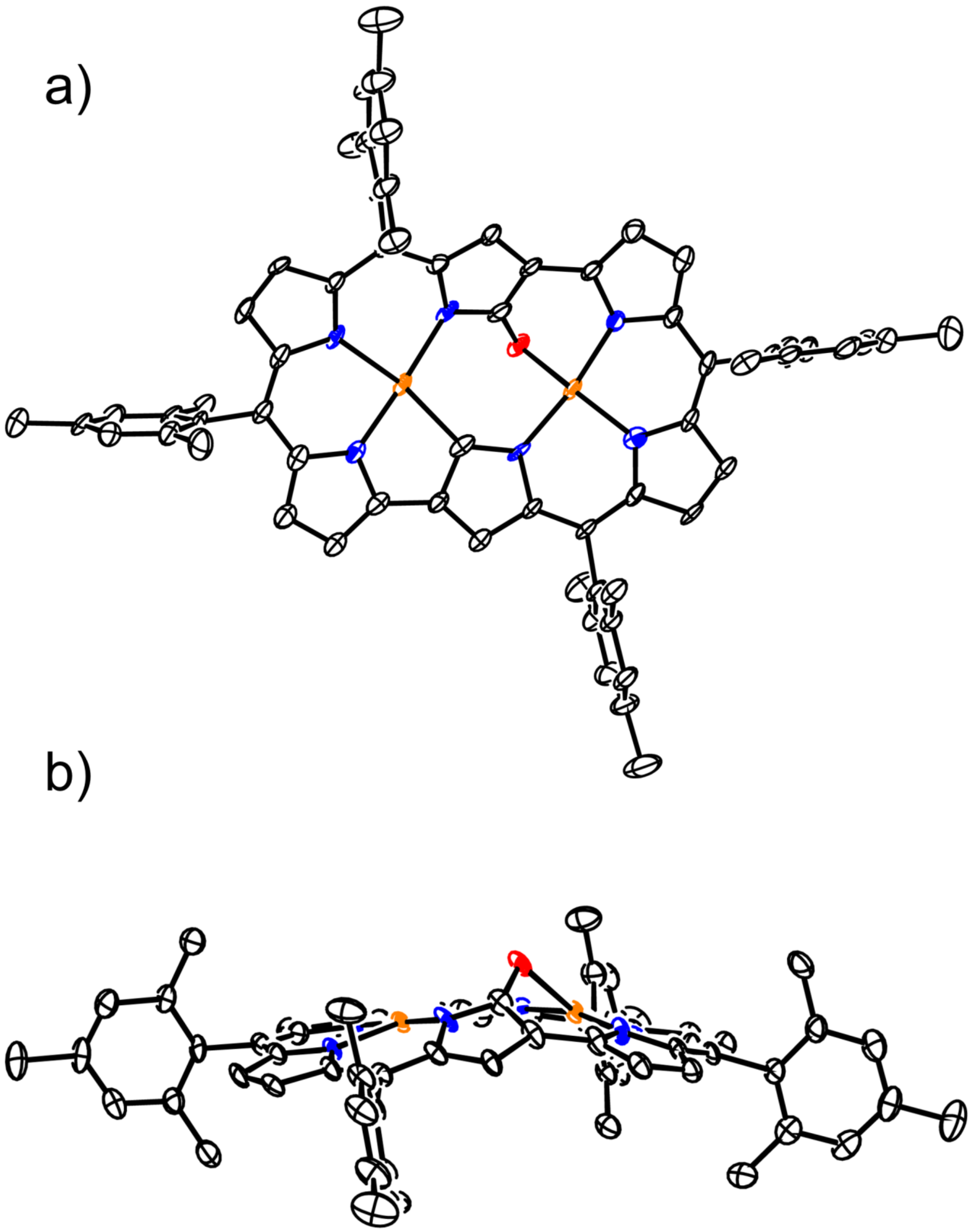

Supplementary Figure 49. X-ray crystal structure of 9Pd. (a) Top view and (b) side view. The thermal ellipsoids are drawn at 30% probability level.

Supplementary Table 9. Crystal data and refinement results for compound **10**.

Identification code	exp_2635_sq	
Empirical formula	C128 H106 N12 Pd4	
Formula weight	2237.84	
Temperature	99.9(4) K	
Wavelength	1.54184 Å	
Crystal system	Triclinic	
Space group	P-1	
Unit cell dimensions	a = 15.9679(12) Å	α= 71.862(7)°.
	b = 20.0928(15) Å	β= 71.330(7)°.
	c = 24.0357(17) Å	γ = 86.857(6)°.
Volume	6934.2(10) $Å^3$	
Z	2	
Density (calculated)	1.072 Mg/m^3	
Absorption coefficient	4.455 mm^{-1}	
F(000)	2284	
Crystal size	0.3 x 0.2 x 0.05 mm^3	
Theta range for data collection	2.041 to 66.600°.	
Index ranges	-12<=h<=19, -23<=k<=23, -28<=l<=28	
Reflections collected	47399	
Independent reflections	24470 [R(int) = 0.1167]	
Completeness to theta = 66.600°	100.0 %	
Absorption correction	Semi-empirical from equivalents	
Max. and min. transmission	1.00000 and 0.25443	
Refinement method	Full-matrix least-squares on F^2	
Data / restraints / parameters	24470 / 0 / 1321	
Goodness-of-fit on F^2	0.960	
Final R indices [I>2sigma(I)]	R1 = 0.0861, wR2 = 0.2075	
R indices (all data)	R1 = 0.1358, wR2 = 0.2438	
Extinction coefficient	n/a	
Largest diff. peak and hole	1.459 and -1.054 e.$Å^{-3}$	
CCDC	2239873	

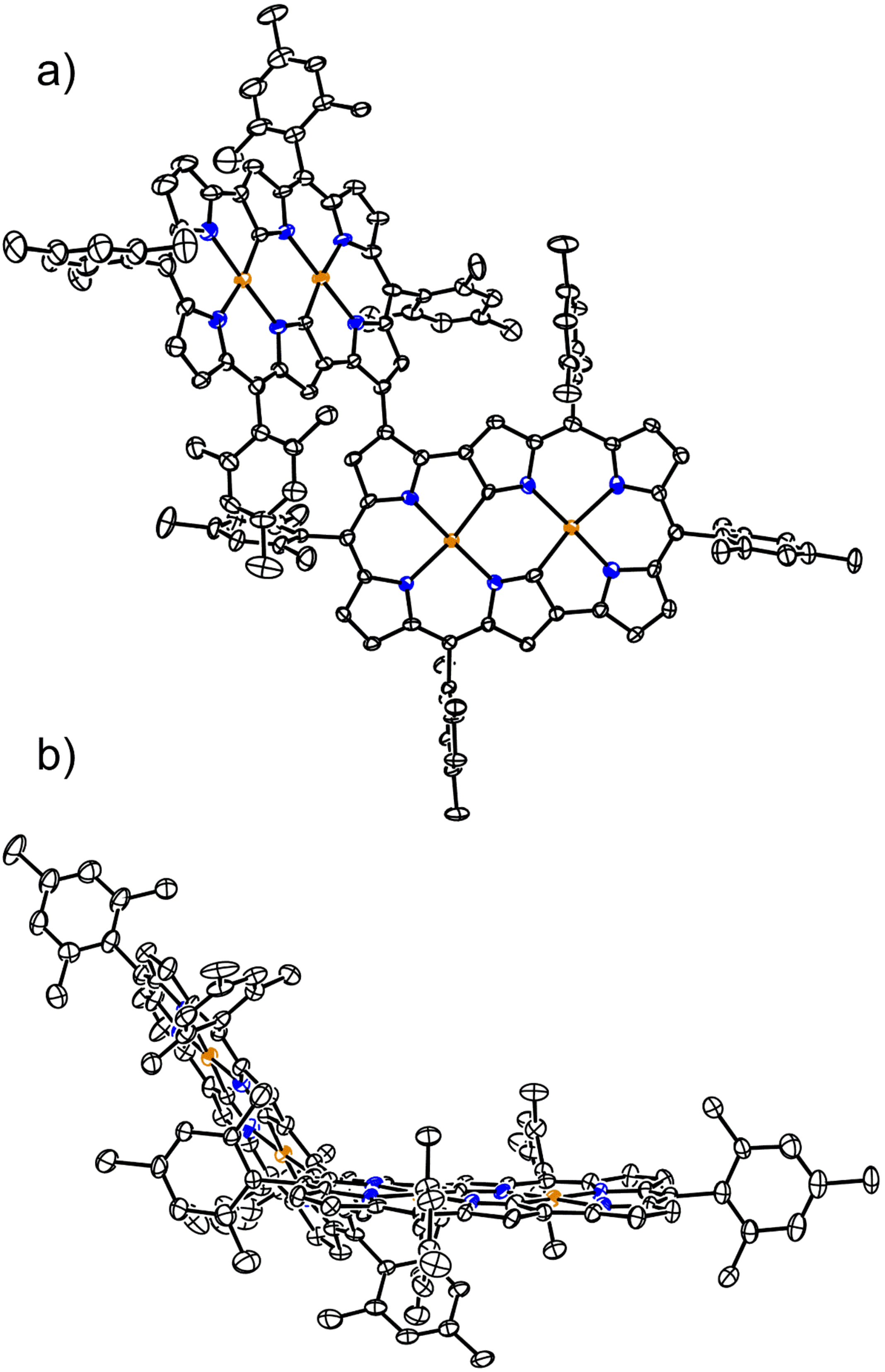

Supplementary Figure 50. X-ray crystal structure of 10. (a) Top view and (b) side view. The thermal ellipsoids are drawn at 30% probability level.

Supplementary Table 10. Crystal data and refinement results for compound **12**.

Identification code	exp_2776_sq	
Empirical formula	C188 H194 N14 Ni2 Pd2	
Formula weight	2979.78	
Temperature	101(1) K	
Wavelength	1.54184 Å	
Crystal system	Triclinic	
Space group	P-1	
Unit cell dimensions	a = 14.3028(4) Å	α= 100.119(2)°.
	b = 17.0485(5) Å	β= 108.070(2)°.
	c = 22.8204(6) Å	γ = 103.957(2)°.
Volume	4941.1(2) $Å^3$	
Z	1	
Density (calculated)	1.001 Mg/m^3	
Absorption coefficient	2.011 mm^{-1}	
F(000)	1568	
Crystal size	0.3 x 0.3 x 0.1 mm^3	
Theta range for data collection	2.775 to 66.601°.	
Index ranges	-17<=h<=15, -20<=k<=20, -18<=l<=27	
Reflections collected	33318	
Independent reflections	17419 [R(int) = 0.0765]	
Completeness to theta = 66.601°	99.9 %	
Absorption correction	Semi-empirical from equivalents	
Max. and min. transmission	1.00000 and 0.72912	
Refinement method	Full-matrix least-squares on F^2	
Data / restraints / parameters	17419 / 66 / 982	
Goodness-of-fit on F^2	1.065	
Final R indices [I>2sigma(I)]	R1 = 0.1079, wR2 = 0.2857	
R indices (all data)	R1 = 0.1182, wR2 = 0.2932	
Extinction coefficient	n/a	
Largest diff. peak and hole	2.041 and -0.942 e.$Å^{-3}$	
CCDC	2239874	

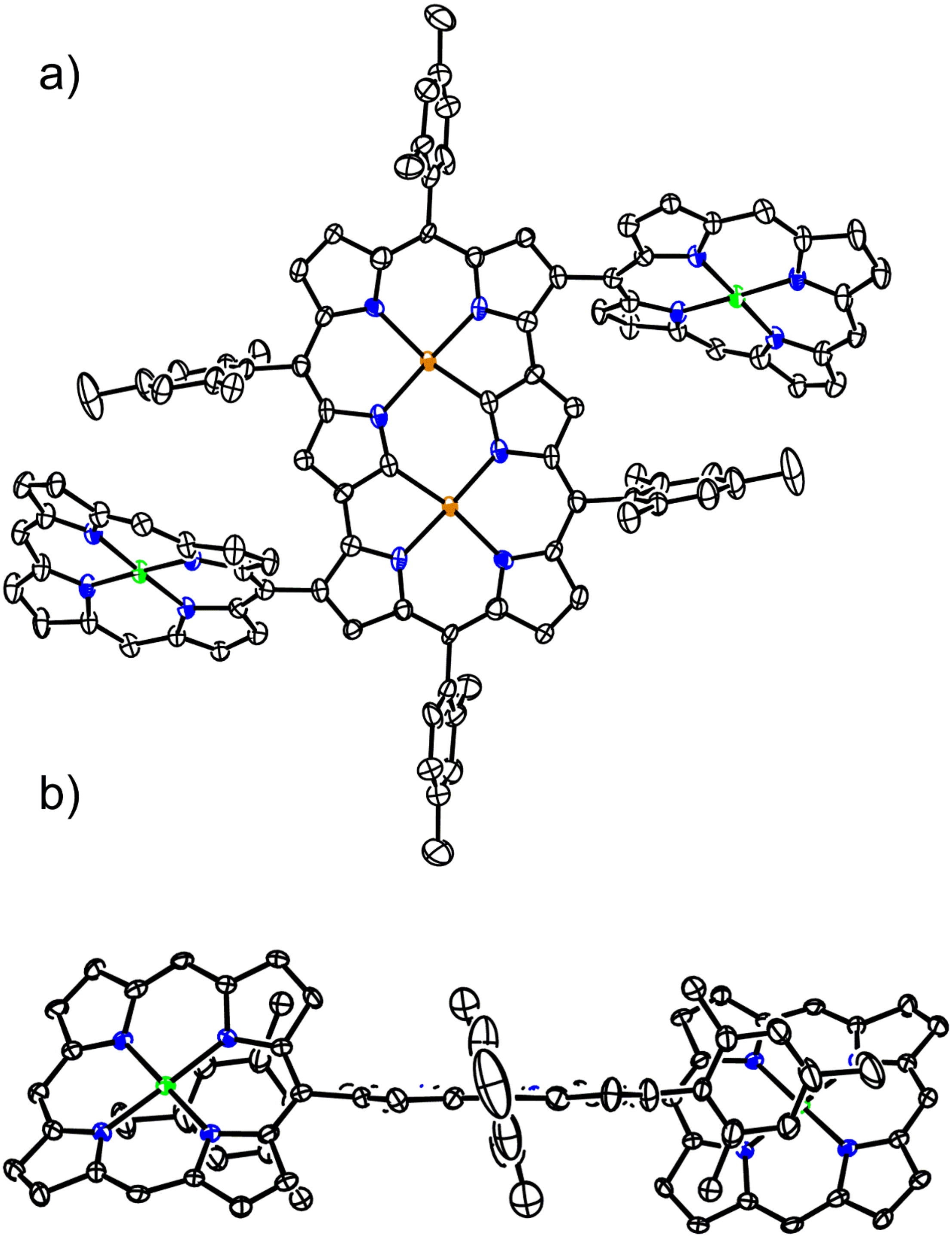

Supplementary Figure 51. X-ray crystal structure of 12. (a) Top view and (b) side view. The thermal ellipsoids are drawn at 30% probability level. *Meso*-substituents of porphyrin parts are omitted for clarity.

DFT Calculation

All calculations were carried out using the Gaussian 09 D.01 program.[1] All structures were fully optimized without any symmetry restriction. All geometries were optimized with the crystal structures as the starting structure at the density functional theory (DFT) method with restricted B3LYP (Becke's three parameter hybrid exchange functionals and the Lee-Yang-Parr correlation functional) level,[2,3] employing 6-311G(d) basis set. Additionally, in order to quantify the impact of the geometric change on aromaticity, the nucleus independent chemical shifts (NICS) values were obtained with the GIAO method based on the final optimized structures. Results for NICS(0) were collected.

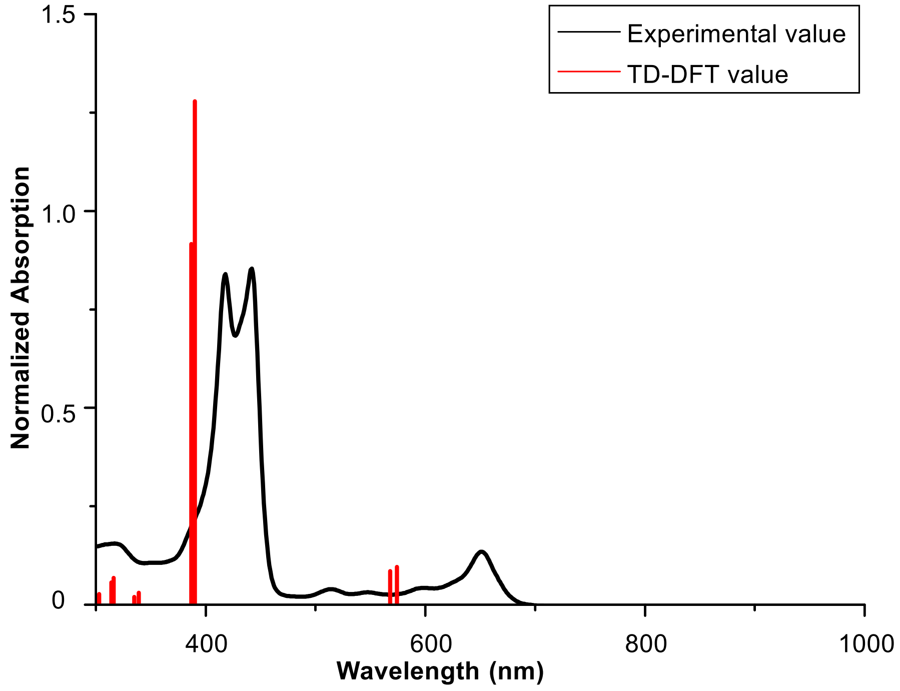

Wavelength (nm)	Oscillator Strengths	Major Transitions
574	0.0956	HOMO→LUMO (58.3%); HOMO→LUMO+1 (18.1%); HOMO-1→LUMO+1 (15.2%); HOMO-1→LUMO (8.3%)
568	0.085	HOMO→LUMO+1 (54.3%); HOMO→LUMO (22.0%); HOMO-1→LUMO (19.1%)
390	1.2783	HOMO-1→LUMO (69.8%); HOMO→LUMO+1 (27.3%)
387	0.9157	HOMO-1→LUMO+1 (77.2%); HOMO→LUMO (19.7%)
339	0.0306	HOMO→LUMO+2 (84.5%); HOMO-3→LUMO+1 (5.8%)
335	0.0197	HOMO-8→LUMO (37.7%); HOMO-3→LUMO+1 (13.3%); HOMO-13→LUMO (12.0%); HOMO-2→LUMO+1 (8.4%)
316	0.0683	HOMO-6→LUMO+1 (49.2%); HOMO-7→LUMO (20.2%); HOMO→LUMO+3 (5.8%); HOMO-8→LUMO+1 (5.6%)
314	0.0569	HOMO-6→LUMO (53.8%); HOMO-7→LUMO+1 (10.9%); HOMO-8→LUMO (9.6%); HOMO→LUMO+3 (6.6%)
303	0.0269	HOMO-8→L+1 (23.9%); HOMO-6→L+1 (18.3%); HOMO-7→L (15.1%); HOMO→LUMO+7 (8.6%)

Supplementary Figure 52. Calculated vertical transitions and major transitions of 6. Calculated by TD-DFT using B3LYP employing the 6-311G(d) basis set.

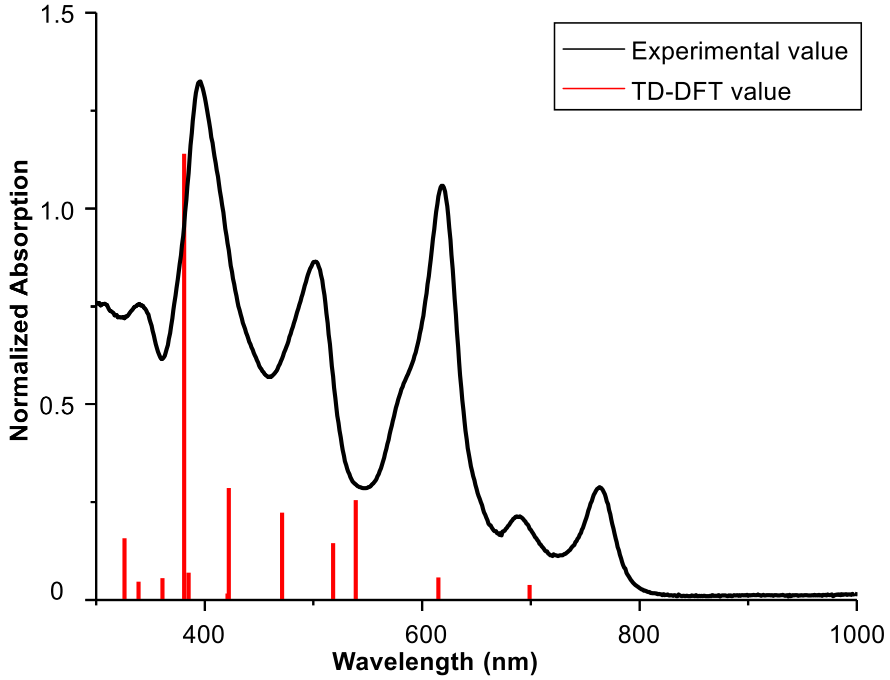

Wavelength (nm)	Oscillator Strengths	Major Transitions
699	0.0388	HOMO→LUMO+1 (68.5%); HOMO-1→LUMO (27.4%)
615	0.0574	HOMO-1→LUMO (35.8%); HOMO-2→LUMO (34.0%); HOMO-3→LUMO (16.2%); HOMO→LUMO+1 (11.3%)
539	0.2547	HOMO-2→LUMO (52.5%); HOMO→LUMO+1 (15.0%); HOMO-1→LUMO (14.5%); HOMO-3→LUMO (7.4%)
518	0.1448	HOMO-3→LUMO (63.3%); HOMO-1→LUMO (18.3%); HOMO→LUMO+2 (11.6%)
471	0.2227	HOMO→LUMO+2 (57.4%); HOMO-4→LUMO+1 (23.2%); HOMO-3→LUMO (7.1%); HOMO-6→LUMO+1 (5.8%)
422	0.2866	HOMO-4→LUMO+1 (53.3%); HOMO-6→LUMO+1 (14.7%); HOMO-16→LUMO (9.3%); HOMO→LUMO+2 (8.2%)
421	0.0163	HOMO-14→LUMO (89.4%)
385	0.0696	HOMO-6→LUMO+1 (60.3%); HOMO-16→LUMO (30.1%)
381	1.1403	HOMO-16→LUMO (54.1%); HOMO-6→LUMO+1 (12.9%); HOMO→LUMO+2 (12.3%); HOMO-4→LUMO+1 (7.0%)
361	0.0561	HOMO-18→LUMO (91.0%)
339	0.0464	HOMO-4→LUMO+2 (81.3%); HOMO-2→LUMO+3 (7.1%)
326	0.1578	HOMO-1→LUMO+3 (76.5%); HOMO-17→LUMO+1 (7.9%)

Supplementary Figure 53. **Calculated vertical transitions and major transitions of 8.** Calculated by TD-DFT using B3LYP employing the 6-311G(d) basis set.

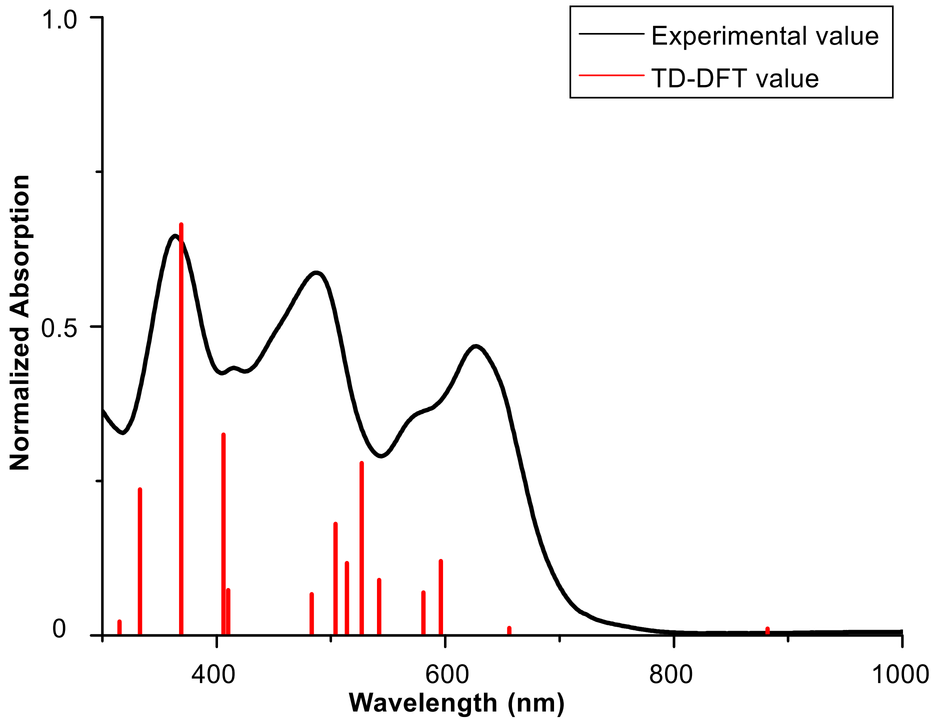

Wavelength (nm)	Oscillator Strengths	Major Transitions
882	0.0111	HOMO-1→LUMO (90.9%)
656	0.0122	HOMO-2→LUMO (57.6%); HOMO→LUMO+1 (34.9%)
596	0.1203	HOMO-4→LUMO (36.1%); HOMO→LUMO+1 (20.7%); HOMO-2→LUMO (18.5%); HOMO→LUMO+2 (18.1%)
581	0.0694	HOMO-4→LUMO (36.6%); HOMO-5→LUMO (34.0%)
542	0.0896	HOMO-5→LUMO (35.2%); HOMO-9→LUMO (17.6%); HOMO-10→LUMO (10.5%); HOMO→LUMO+1 (8.3%)
527	0.2788	HOMO→LUMO+2 (43.3%); HOMO-9→LUMO (9.5%)
514	0.1169	HOMO-1→LUMO+1 (33.2%); HOMO→LUMO+2 1(7.4%); HOMO-5→LUMO (16.1%); HOMO-4→LUMO (6.8%)
504	0.1806	HOMO-1→LUMO+1 (48.1%); HOMO-9→LUMO (10.3%)
483	0.0668	HOMO-11→LUMO (49.9%); HOMO-13→LUMO (14.8%)
410	0.0734	HOMO→LUMO+3 (59.0%); HOMO-4→LUMO+1 (13.6%)
406	0.3246	HOMO-1→LUMO+2 (29.5%); HOMO-14→LUMO (10.7%); HOMO-3→LUMO+2 (10.2%); HOMO-18→LUMO (10.0%)
369	0.6651	HOMO-4→LUMO+1 (21.0%); HOMO-5→LUMO+1 (17.7%); HOMO-9→LUMO+1 (10.4%); HOMO→LUMO+3 (7.2%)
333	0.236	HOMO-5→LUMO+2 (43.4%); HOMO-22→LUMO (13.3%)
315	0.0223	HOMO-14→LUMO+1 (21.7%); HOMO→LUMO+5 (16.4%); HOMO-24→LUMO (15.0%); HOMO-25→LUMO (10.2%)

Supplementary Figure 54. **Calculated vertical transitions and major transitions of 9.** Calculated by TD-DFT using B3LYP employing the 6-311G(d) basis set.

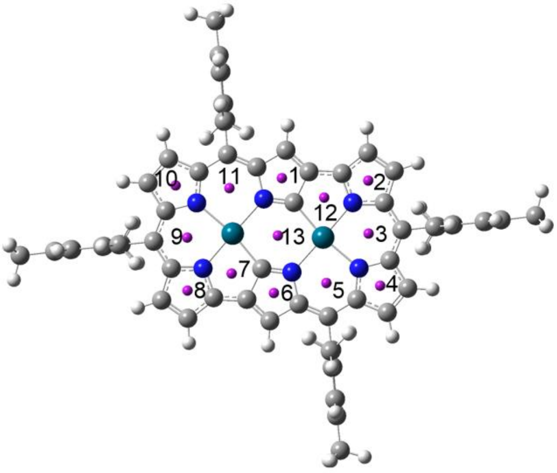

Supplementary Figure 55. **Calculated NICS(0) of 8.** Purple dots indicate Bq atoms.

	**1**	**2**	**3**	**4**	**5**	**6**	**7**
**NICS**	13.2469	-4.6689	14.6965	-5.7466	8.9518	13.0151	11.5915
	**8**	**9**	**10**	**11**	**12**	**13**	
**NICS**	-4.5361	14.5440	- 5.6966	8.9791	11.8668	5.4134	

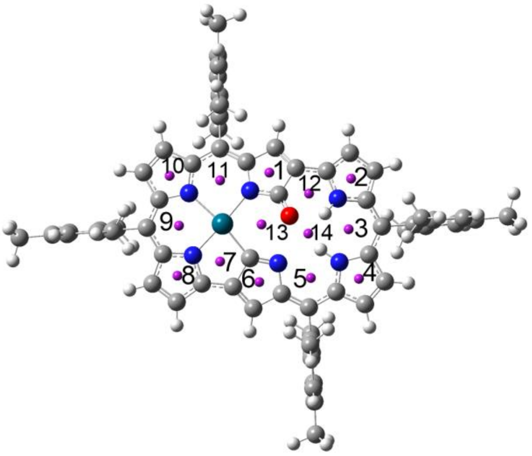

Supplementary Figure 56. **Calculated NICS(0) of 9.** Purple dots indicate Bq atoms.

	**1**	**2**	**3**	**4**	**5**	**6**	**7**
**NICS**	20.9202	-3.7810	19.6890	-1.7119	19.6248	33.3647	24.8369
	8	9	10	11	12	13	14
**NICS**	-11.390	25.356	-9.8456	17.0684	16.6577	10.4340	13.8865

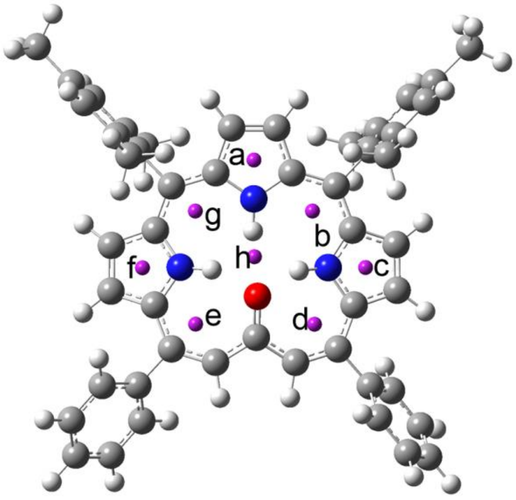

Supplementary Figure 57. Calculated NICS(0) of 6. Purple dots indicate Bq atoms.

	**a**	**b**	**c**	**d**	**e**	**f**	**g**	**h**
**NICS**	-11.9411	-17.8101	-13.9625	-17.5335	-17.8287	-14.3612	-18.1181	-12.1483

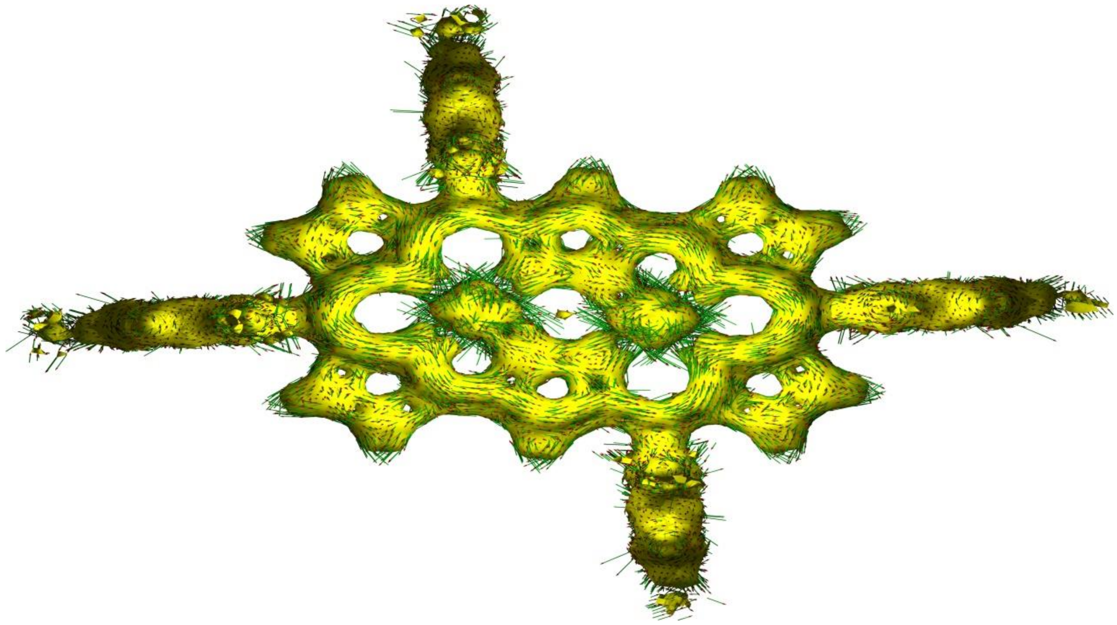

Supplementary Figure 58. **ACID plot of 8 at isosurface value of 0.04.** The external magnetic field was applied in the direction from the back of the paper to the surface.

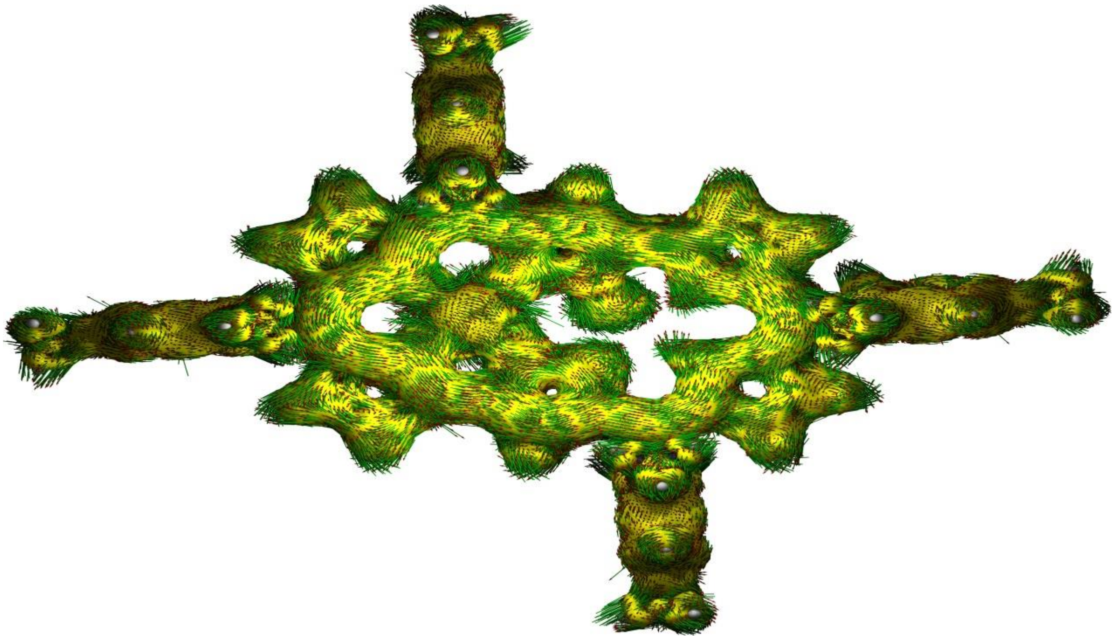

Supplementary Figure 59. **ACID plot of 9 at isosurface value of 0.04.** The external magnetic field was applied in the direction from the back of the paper to the surface.

[1] Gaussian 09, Revision C.01, M. J. Frisch, G. W. Trucks, H. B. Schlegel, G. E. Scuseria, M. A. Robb, J. R. Cheeseman, G. Scalmani, V. Barone, B. Mennucci, G. A. Petersson, H. Nakatsuji, M. Caricato, X. Li, H. P. Hratchian, A. F. Izmaylov, J. Bloino, G. Zheng, J. L. Sonnenberg, M. Hada, M. Ehara, K. Toyota, R. Fukuda, J. Hasegawa, M. Ishida, T. Nakajima, Y. Honda, O. Kitao, H. Nakai, T. Vreven, J. A. Montgomery, Jr., J. E. Peralta, F. Ogliaro, M. Bearpark, J. J. Heyd, E. Brothers, K. N. Kudin, V. N. Staroverov, R. Kobayashi, J. Normand, K. Raghavachari, A. Rendell, J. C. Burant, S. S. Iyengar, J. Tomasi, M. Cossi, N. Rega, J. M. Millam, M. Klene, J. E. Knox, J. B. Cross, V. Bakken, C. Adamo, J. Jaramillo, R. Gomperts, R. E. Stratmann, O. Yazyev, A. J. Austin, R. Cammi, C. Pomelli, J. W. Ochterski, R. L. Martin, K. Morokuma, V. G. Zakrzewski, G. A. Voth, P. Salvador, J. J. Dannenberg, S. Dapprich, A. D. Daniels, Ö. Farkas, J. B. Foresman, J. V. Ortiz, J. Cioslowski, D. J. Fox, Gaussian, Inc., Wallingford CT, **2009**.

[2] A. D. Becke, *J. Chem. Phys.* **1993**, *98*, 1372-1377.

[3] C. Lee, W. Yang and R. G. Parr, *Phys. Rev. B* **1998**, *37*, 785-789.
